# Carbon Dioxide Electroreduction on Gold without Metal or Organic Cations

**DOI:** 10.1021/acscatal.5c02785

**Published:** 2025-06-18

**Authors:** Hansaem Jang, Ciarán O’Brien, Nathaniel J. D. Hill, Adrian M. Gardner, Ivan Scivetti, Gilberto Teobaldi, Alexander J. Cowan

**Affiliations:** † Stephenson Institute for Renewable Energy (SIRE) and the Department of Chemistry, 4591 University of Liverpool, Liverpool L69 7ZF, United Kingdom; ‡ Scientific Computing Department, Rutherford Appleton Laboratory, STFC UKRI, Harwell Campus, Didcot OX11 0QX, United Kingdom; § Early Career Laser Laboratory and Surface Science Research Centre, 4591 University of Liverpool, Liverpool L69 3BX, United Kingdom; ¶ Scientific Computing Department, Daresbury Laboratory, STFC UKRI, Daresbury, Warrington WA4 4AD, United Kingdom; ∥ Central Laser Facility, Research Complex at Harwell, STFC-Rutherford Appleton Laboratory, Harwell Campus, Didcot OX11 0QX, United Kingdom

**Keywords:** CO_2_R, eCO_2_R, SFG, VSFG, DFT

## Abstract

Extensive research efforts have been concentrated into the conversion of CO_2_ into value-added chemicals as it provides a route to a circular carbon economy. Electroreduction of CO_2_ on Au surfaces allows for the selective transformation of CO_2_ into CO via carbon dioxide reduction reaction (CO_2_RR), and the catalytic activity depends on the concentration and identity of cations present at the electrode–electrolyte interface. Experimental reports performed under typical CO_2_RR-operating conditions have widely shown that the CO_2_RR is enabled by the presence of metal or organic cations in the cathodic interfacial microenvironment. A remaining question is to address if CO_2_RR can occur in the absence of metal or organic cations and, if so, what the mechanism may be. Here, we show that CO_2_ can be electrochemically reduced to CO on Au in acidic electrolytes rigorously controlled to avoid the presence of metal and organic cations and systematically suggest the important contributions allowing this reaction to proceed. The formation of CO is confirmed by both qualitative and quantitative methods using potentiodynamic CO-stripping scans and chromatography-assisted constant potential electrolysis. Calculations indicate that H_3_O^+^ is able to stabilize the formation of *CO_2_^–^, albeit at more negative potentials than when an alkali metal cation is present. Spectroelectrochemical experiments show that the electric field at the interface is reduced when metal cations are not added, indicating that the decreased field stabilization of intermediates could play an important role in increased overpotential required for the CO_2_RR to occur.

## Graphical abstract

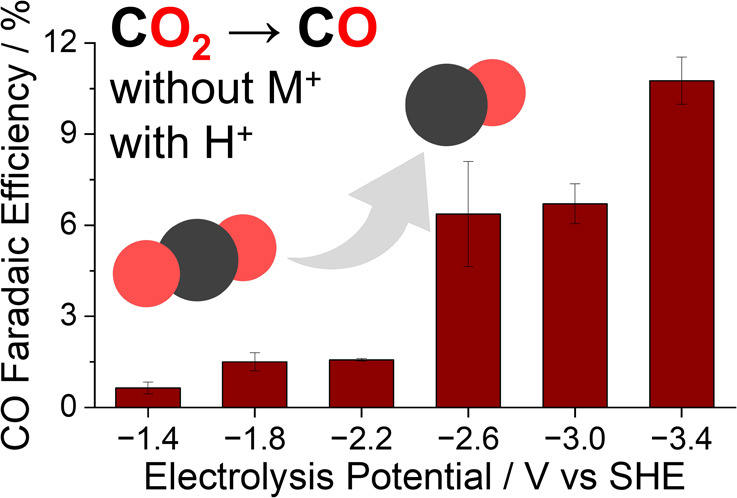

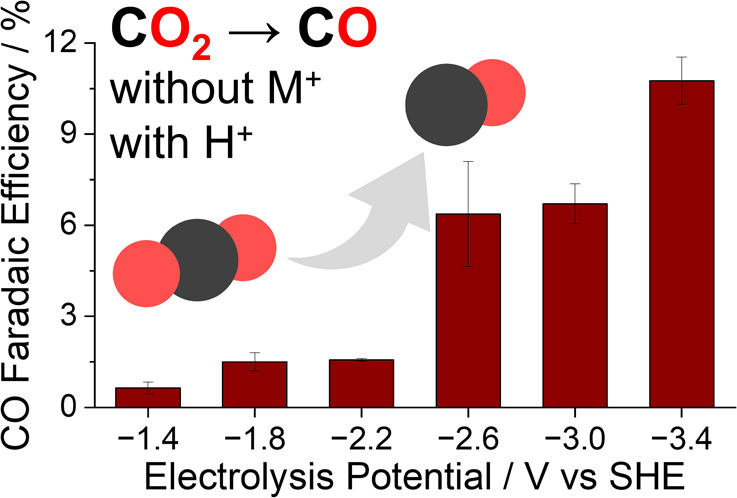

## Introduction

Carbon dioxide can be electrochemically reduced into useful chemicals.^1^ As the carbon dioxide reduction reaction (CO_2_RR) occurs at the electrode–electrolyte interface, the CO_2_RR activity and selectivity are governed by the interfacial microenvironment.^2,3^ The microenvironment can be modulated by tuning the structural properties of electrode surfaces.^4–6^ Importantly, it can also be modified by the species present within the double layer of the negatively charged working electrode surface. Cations that can donate protons (e.g., H_3_O^+^) can participate in the hydrogen evolution reaction (HER), which is a major competing reaction against the CO_2_RR on the cathode.^7^ On the other hand, cations that can maintain their positive charge at the interface (i.e., metal and organic cations) can contribute to the CO_2_RR performance, which is known as the “cation effect”. The main theories regarding the mechanisms of cation effects are (i) electrostatic stabilization of intermediates by interfacial species, (ii) coordinative stabilization of intermediates by direct interaction between the cation and the intermediate, (iii) buffering the local pH, (iv) offering a local hydrophobic microenvironment by modulating the water structure, and (v) modulating the electronic structure of adsorbed intermediates by the adjacent specifically adsorbed cation.^8–10^

When metal or organic cations are absent from the cathode surface, CO_2_RR is reported to not occur under typical operating conditions.^10,11^ Koper and co-workers showed that the presence of metal cations on Ag, Au, and Cu surfaces results in a favorable local electrostatic interaction between a partially solvated metal cation and CO_2_, thereby stabilizing the adsorbed CO_2_ intermediates (*CO_2_^–^; clarification regarding notations available in Note S1); in the absence of these cations, the barrier to CO_2_RR is difficult to overcome on the studied surfaces.^11^ Subsequently, it has been demonstrated that organic cations, and other molecular entities that can impart cationic functionalities to the cathode surface, can also facilitate CO_2_RR.^12–17^ On the basis of these reports, it is clear that metal and organic cations can stabilize intermediates; however, there is some debate on how this occurs—whether it is electrostatic stabilization or coordinative stabilization. It may be that the electric field alone could be enough to lead to the occurrence of CO_2_RR.

A small number of reports have emerged over recent years regarding the occurrence of CO_2_RR in the absence of metal or organic cations. In contrast to their earlier work,^11^ Koper and co-workers reported that CO_2_ reduction to CO can take place in the absence of cations on a monolayer of Pd deposited on a Pt(111) single crystal and attributed the disparity to the nature of Pd which has a relatively stronger binding to CO and thus a lower activation barrier for CO formation.^18^ Gu and co-workers reported that CO_2_ was reduced into CO on Ag without any metal cations, but not on Au, and attributed this disparity to the difference in potential of zero charge (PZC) values.^19^ Their interpretation is based on a change in the effective charge distribution at the electrode–electrolyte interface caused by electrostatic interactions between metal cations and cathode depending on the applied potential with respect to the PZC (cf. Note S2 for a detailed discussion). Notably, there are reports that Ni single atom catalysts,^20,21^ unlike bulk state Ni,^22^ can catalyze the CO_2_RR in the absence of metal or organic cations. In addition, it has also been reported that CO_2_RR occurs on some metal catalysts without adding extraneous cations when the transport of protons in the microenvironment is regulated.^23–25^ However, the origin of how CO_2_RR can occur without metal or organic cations, and the systematic study of how to enable CO_2_RR in the absence of these cations, still remains elusive.

In this work, we show that CO_2_RR can occur on conventional polycrystalline Au surfaces in acidic electrolytes rigorously controlled to avoid the presence of metal or organic cations at potentials negative of −1.4 V versus the standard hydrogen electrode (SHE; hereinafter, all potentials are referenced against SHE). The electroreduction of CO_2_ into CO under these conditions is confirmed by electroanalysis and chromatography. Computational modeling is implemented to estimate whether CO_2_RR intermediates can be formed under these conditions. Density functional theory (DFT) calculations suggest that the formation of CO_2_RR intermediates can take place in the absence of metal or organic cations with H_3_O^+^ able to stabilize the initially formed *CO_2_^–^. Surface-sensitive vibrational spectroscopy is performed as a measure of relative field strengths at the electrode–electrolyte interface. Vibrational sum frequency generation (VSFG) reveals that the potential-dependent frequency shift of CO reporter molecules on the Au surface is increased by adding metal cations (1 mM K_2_SO_4_). This is indicative of increased local electric field strength, which is likely to be a cause of the difference in overpotentials required for CO_2_RR in the presence and absence of these additional metal cations.

## Results and Discussion

### Potentiodynamic Electroanalysis

Cyclic voltammetry (CV) with varied potential windows can offer qualitative evidence related to the occurrence of CO_2_RR.^11,17,26^ In Figure 1, we perform CV in ultrapure H_2_SO_4_ solution without agitating the electrolyte, scanning from the open circuit potential (OCP) to a potential minimum (*E*_min_), to +1.1 V, and then back to the OCP at 50 mV s^–1^. If CO is produced during the forward scan (from OCP to *E*_min_), then the current will rise as a consequence of CO stripping during the reverse scan (from *E*_min_ to +1.1 V). Here, an *E*_min_ is varied with a potential difference of 0.4 V.

**Figure 1:**
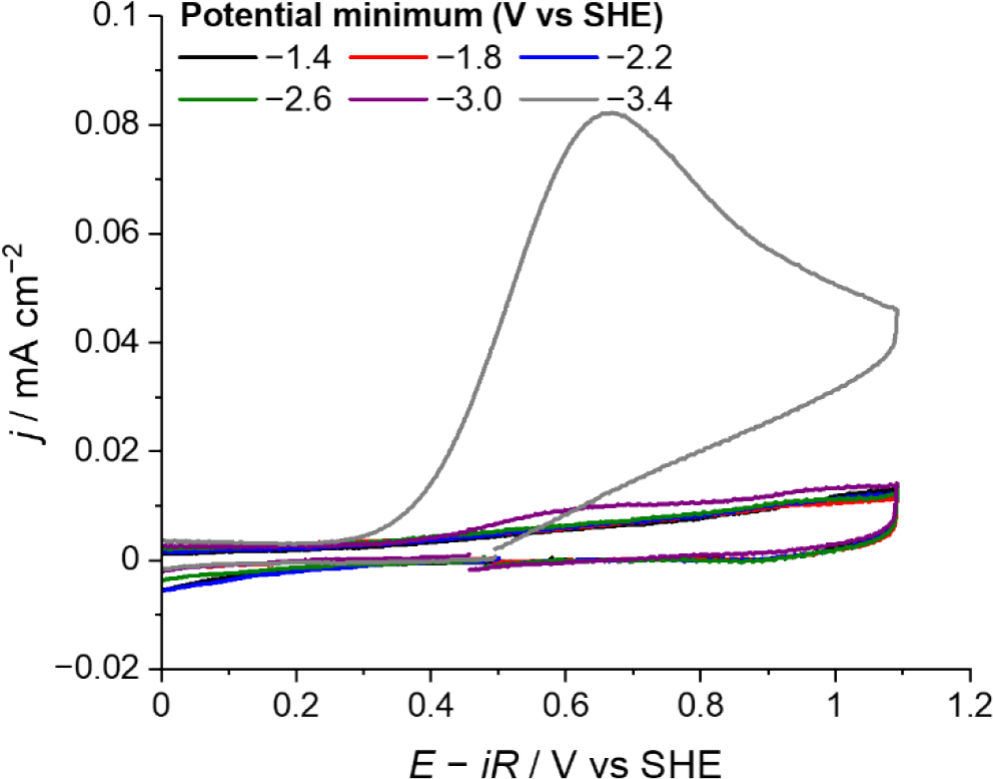
Cyclic voltammograms obtained on the Au electrode in CO_2_-saturated 1 mM H_2_SO_4_ solution. The electrode potential was initially scanned from the OCP to the potential minimum as stated in the legend, and then to +1.1 V, and back to the OCP at 50 mV s^–1^. The full window voltammograms are available in Figures S1–S6.

The characteristic CO stripping peak appears when *E*_min_ is at a potential of −3.0 V or more negative (Figure 1 and Figures S1–S6). The CO stripping peak becomes pronounced when the limiting potential is −3.4 V even after a single CV cycle. Control experiments under Ar (Figure S7) confirms that CO formation is a result of application of negative potentials. However, there exists a possibility that the CO_2_RR arises from adventitious and/or autogenous cationic impurities. We discard the possibility that adventitious cations act on the activation of CO_2_RR based on three reasons. First, the concentration of adventitious cations in the electrolyte used in this study was below the limit of detection (Table S1). Second, it has been reported that the CO_2_RR remains inactive on Au surfaces in the presence of potential adventitious cation impurities together with deliberately added [K^+^] of 200 nM,^17^ and the metal cation concentration of the electrolyte used in our work is far less than the aforementioned report. Third, CO_2_RR still occurs in the presence of chelating agents in the electrolyte (*vide infra*).

We consider the possibility that autogenous cations that are produced in situ during the electrolysis contribute to the occurrence of CO_2_RR. While HER and CO_2_RR are occurring on the cathode, oxidative reactions would proceed on the anode (i.e., counter electrode), which can result in the formation of metal cations and/or cationic complexes as a result of anodic dissolution.^27–30^ These cations can be electrostatically attracted to the negatively charged cathode where they can be electrodeposited. Upon approaching to the cathode surface, the cations that are in the interfacial microenvironment could possibly give rise to the cation effect, and hence, the CO_2_RR. To circumvent the inflow of autogenous cations from the counter electrode to the working electrode surface, we separated the Au counter electrode with a porous polytetrafluoroethylene membrane and then filled the counter chamber with Au ion scavengers. Despite the introduction of scavengers, CO_2_ was still electrochemically reduced to CO (Figure 2 and Figure S8). Therefore, it is unlikely that the CO_2_RR is a consequence of autogenous cations arising from the counter electrode.

**Figure 2:**
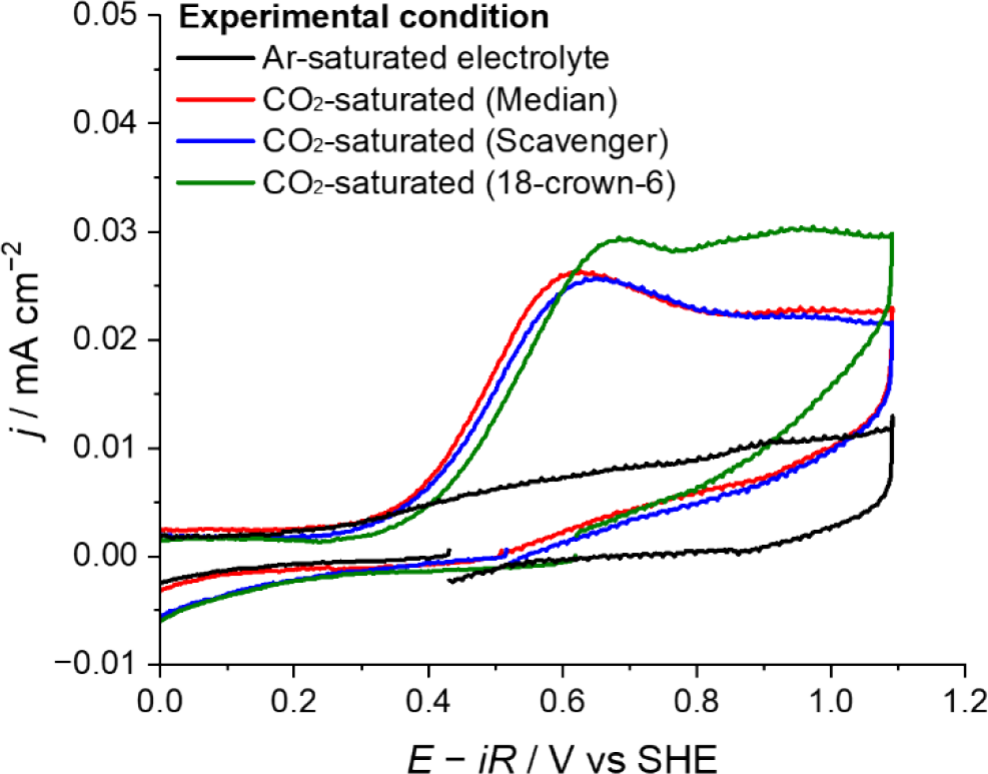
Cyclic voltammograms obtained on the Au electrode in CO_2_-saturated 1 mM H_2_SO_4_ solution under conditions stated in the legend. The electrode potential was scanned from the OCP to −3.4 V, and then to +1.1 V, and back to the OCP at 50 mV s^–1^.

Autogenous cations may also be transiently generated on cathode. Buonsanti and co-workers detected the formation of transient Cu^+^ ions on metallic Cu cathode via chelation during the CO_2_RR at negative potentials where Cu falls within the immunity region in the Pourbaix diagram.^31^ We introduced a crown ether to the electrolyte as a chelating agent and performed the electrolysis. Despite the presence of chelators, the electroreduction of CO_2_ took place on the cathode surface (Figure 2). This observation indicates that the occurrence of CO_2_RR is not likely due to the generation of transient cations. Furthermore, even in the absence of chelators, the postelectrolysis analysis results showed that the Au ion concentration after electrolysis remained below the limit of detection (Table S1, Note S3, and Figure S9). Taken together, we concluded that the stabilization of the adsorbed CO_2_ intermediates arises as a result of the negative potentials applied to Au surfaces, rather than the autogenous cations.

### Constant Potential Electrolysis

Measuring CO stripping currents by CV provides a facile initial indicator of CO_2_RR to CO. However, the CV measurement may not be able to identify small quantities of CO_2_RR products. Thus, we have also performed chronoamperometry at a constant potential and quantify the gaseous products using gas chromatography (GC) to enable direct CO measurements.

The CO_2_RR rate on Au is independent of the proton donor environment.^32^ Therefore, the Faradaic efficiency of CO_2_RR on Au should enhance by lessening the proton donor population at the cathode surface. Peters and co-workers showed that a current density of −10 mA cm^–2^ could lead to proton depletion at the interface in pH 2 solution (i.e., [H^+^] ≈ 10 mM).^23^ Given that we used pH 2.73 solutions in this work (i.e., [H^+^] ≈ 1.86 mM), we presume that a current density of −10 mA cm^–2^ or greater will be effective to promote the CO_2_RR at the interface. In pursuit of more negative current densities, we perform electrolysis through chronoamperometry under agitated conditions.

Different from the CV experiments (Figure 1), CO production is observed in all potentials studied in this work, including potentials more positive than −3.0 V, as shown in Figure 3. It is of particular importance that CO was produced when electrolysis was performed at potentials more positive than −1.9 V. The standard potential required to generate a nonadsorbed CO_2_ intermediate (CO_2_^•–^) is −1.9 V.^33^ Alternatively, since solvated electrons have a standard potential equivalent of −2.9 V, the first CO_2_ reduction step can take place in the presence of solvated electrons and the as-formed nonadsorbed CO_2_^•–^ radicals can be readily stabilized on Au surfaces.^34^ Therefore, the detection of CO at potentials positive of −1.9 V can serve as an indicator of the CO_2_RR proceeding via nonradical routes. In Figure 3, the CO production is observed at −1.4 and −1.8 V (cf. Figure S10 confirms that the Faradaic efficiency of the −1.4 V experiment is not a result of any background CO in the GC), which indicates that an unstabilized radical intermediate is not necessarily required for CO_2_RR to occur on Au in our experimental system.

**Figure 3:**
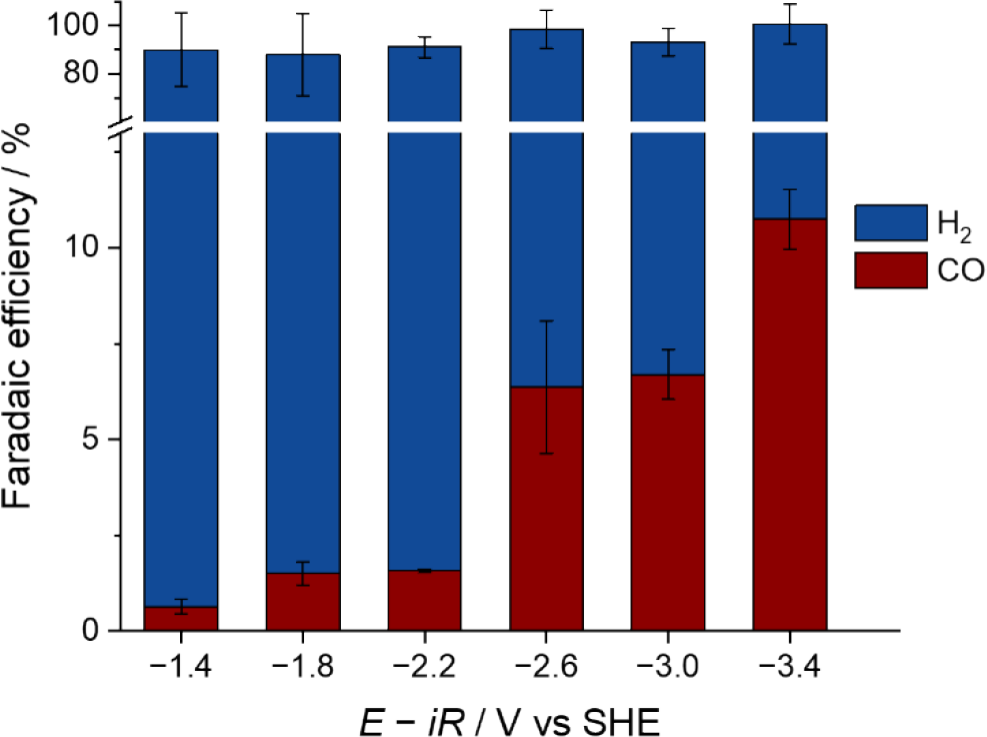
Composition analysis of gas products after bulk electrolysis at different potentials with magnetic stirring at 600 rpm in CO_2_-saturated 1 mM H_2_SO_4_ solution (for details, see Methods).

On the basis of the bulk electrolysis results, we can estimate the CO_2_RR onset potential on Au in electrolytes rigorously controlled to avoid the presence of metal or organic cations as slightly more positive than −1.4 V. In addition, the CO_2_RR becomes more pronounced at the potentials negative of −2.6 V. Given that these potential values are much more negative than conventional values, the difference between this work and the previous reports^11,35^ where the CO_2_RR was not observed on Au can be attributed to the use of different potential windows. On the other hand, in the presence of metal cations such as K^+^ or Cs^+^, an onset potential for CO_2_RR approaches a potential of −0.8 V as the cation concentration increases.^11,17^ Comparison between the two cases indicates that the CO_2_RR can occur irrespective of the presence of metal cations but at significantly greater overpotentials when metal cations are absent.

### Computational Modeling

We have shown that the electroreduction of CO_2_ into CO, and thus the transformation of CO_2_ into the intermediate species, can occur also on Au surfaces without adding metal or organic cations. Why does the discrepancy arise between the literature and our work? To investigate this point, we simulated the electronic structure of CO_2_ in the presence of H_3_O^+^ and K^+^ on a model Au surface.

Insights into the nature of the direct molecular interaction between the cations and the CO_2_ molecule, and its effect on the CO_2_ molecular orbitals relevant to the CO_2_RR, can be gained from the analysis of the projected density of states (PDOS) for simplified interface models. In theorizing a unifying mechanism for the cation-mediated reduction of CO_2_, Shin et al.^36^ have reported that the lowering of the CO_2_ LUMO through interaction with the cation in the vicinity of the surface is the key factor in the transformation of neutral CO_2_ to its chemisorbed, negatively charged intermediate state (*CO_2_^–^). The nature of this interaction is likely due to an interplay between electrostatic effects and shorter range (though not necessarily electrostatic only *a priori*) inner-sphere interactions,^37^ with the relative weight of these components posited to be cation-dependent.

In our simulations of a single CO_2_ molecule on bare Au(111) in the presence of 1 K^+^ (Figure 4a) and 1 H_3_O^+^ (Figure 4b), the calculated LUMO (Figure 4c) of CO_2_ is shown to be lowered with respect to the system in the absence of cations, albeit within the constraints of a solvent-free, “Au(111)” single-crystal surface model. Notably, the magnitude of this negative shift is larger in the presence of K^+^ (1.8 eV) compared to H_3_O^+^ (1.4 eV). It should be noted that in both coadsorbed configurations in Figure 4, CO_2_ remains in a linear geometry and is physisorbed onto the Au surface through dispersive interactions. As the CO_2_ LUMO remains above the Fermi level, no reductive rehybridization takes place to generate *CO_2_^–^. Further permutations of these geometries were modeled, displacing the cation and Au surface in the *z*-direction to different distances from the CO_2_ molecule. This was done to chart the effect of the cation orbital interaction with the CO_2_ LUMO—a detailed description available in Note S4 and Figures S11–S13. From the analysis of the full set of coadsorbed geometries modeled (cf. Figure S13 and Table S2 for more information), we suggest that the primary factor in the lowering of the LUMO can be attributed to the proximity of the C=O bond to the center of positive charge at the cation (with resulting polarization breaking symmetry of the linear CO_2_ molecule).

**Figure 4:**
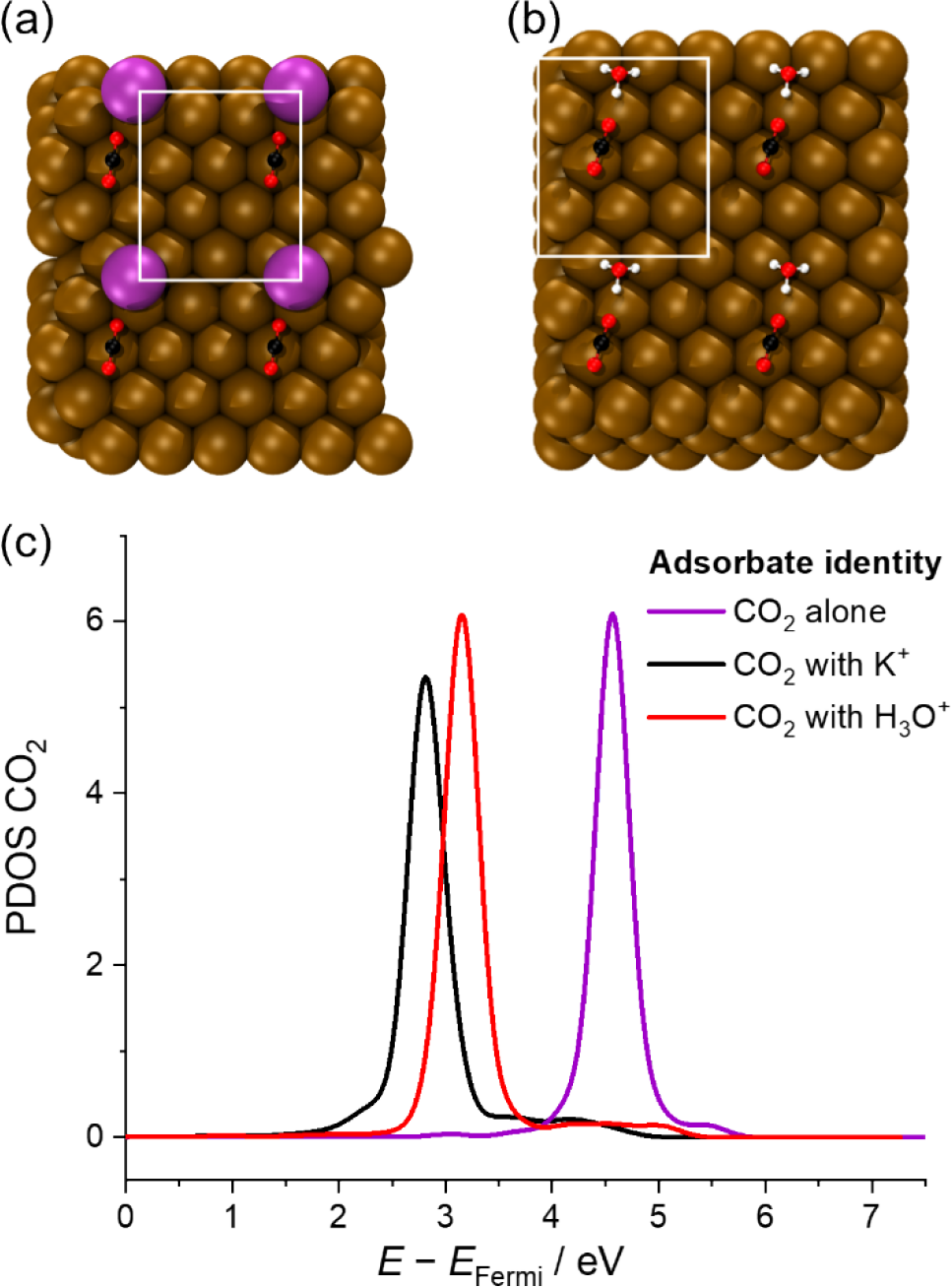
Simulations of experimental models. (a) Relaxed geometry of the CO_2_ coadsorbed with K^+^ (2 × 2 supercells of the simulation cell plotted for ease of visualization; simulation cell *xy*-plane size is bounded in white. (b) Relaxed geometry of the CO_2_ coadsorbed with H_3_O^+^ in the manner of (a). (c) Section of CO_2_ PDOS containing the calculated LUMO of the CO_2_ in the presence and absence of K^+^ or H_3_O^+^ coadsorbates.

Shin et al.^36^ rationalize that the surface charge density of cations, taken as approximate proxy to the Stern layer, is the dominant factor for the rate of reduction. Thus, increasing the surface charge for equivalently sized periodic slabs of Au(111) should therefore result in the same transformation of CO_2_ to *CO_2_^–^ past some threshold charge density, irrespective of cation identity. To investigate this hypothesis, we have built simplified interface models with increased excess charge densities at the Au slab, for which an extra cation (i.e., two cations per simulation cell) was included to lower the CO_2_ LUMO below the Fermi level. A sparse coating of interfacial water was also included to model solvation of the CO_2_ and cations, and to screen the resultant highly localized positive charge on the cations (cf. Figure 5a for model visuals).

**Figure 5:**
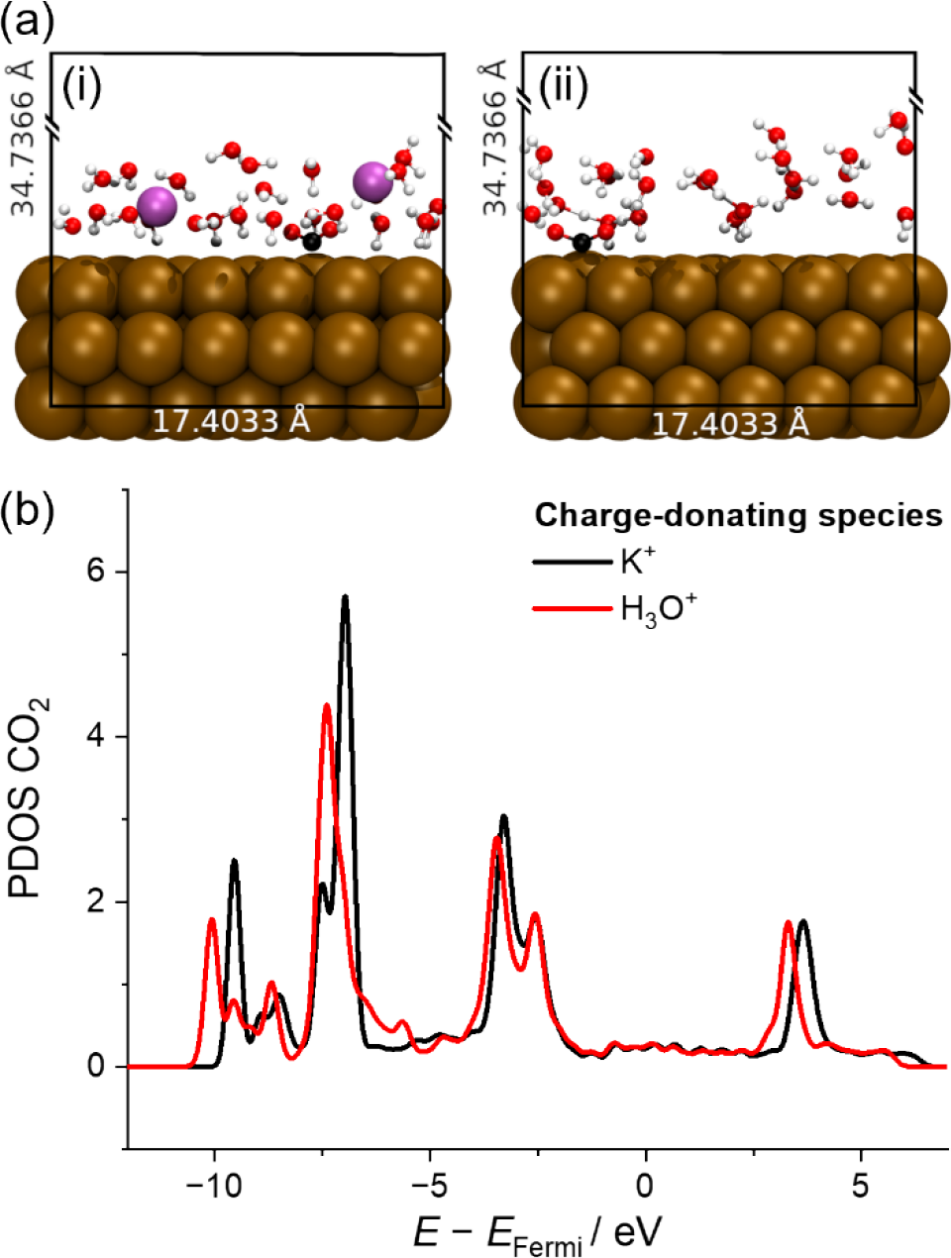
Simulations of experimental models. (a) Simulation cells containing (i) 2× K^+^ and (ii) 2× H_3_O^+^ in a water layer above a negatively charged slab of Au(111) and a chemisorbed CO_2_ molecule. Vacuum layers in both simulation cells are truncated in the figure for visual clarity. (b) PDOS of CO_2_ molecules in (i) and (ii) pictures.

Results of a Bader charge analysis of both system snapshots in Figure 5 are given in Table 1. The “cationic” species indeed lose electronic charge approaching 1 e^–^ (per cation) from their initial atomic charges. Analogously, the Au slab retains an excess charge of approximately 1 e^–^ and the CO_2_ molecule abstracts (0.5–0.7 e^–^), which qualitatively confirms our working hypothesis on the leading role of the electrode surface charge regardless of the cationic nature. Each model system necessarily remains charge-neutral overall. However, as the population of protons at the interface is maintained at a potential-dependent steady state by the HER and the transport of cationic species to the Stern layer, our model does not represent a realistic concentration of H^+^ nor that of interfacial water. We reiterate the present models to illustrate the interplay between short-ranged electrostatics and cation–CO_2_ rehybridization for the energy of the CO_2_ LUMO with respect to the metal Fermi level (*E*_Fermi_), in turn taken as approximate proxy for the ease of CO_2_ activation to *CO_2_.

**Table 1:** Charge Transfer^a^ of Species in Slab-Charging Simulations Quantified by Bader Charge Analysis

system^b^	charge transferred from alkali cations	charge transfer to CO_2_	charge transfer to Au(111)	charge transfer of water layer^c^
(2× K^+^)	–1.79 e^–^	+0.61 e^–^	+0.82 e^–^	+0.35 e^–^
(2× H_3_O^+^)	N/A	+0.71 e^–^	+0.78 e^–^	–1.49 e^–^

a Calculated using (*Q*_Bader_ – *Q*_atomic_).

b With the copresence of (1× CO_2_) using 4 × 3 × 3 Au­(111_orthorhombic_).

c (24× H_2_O).

Quantitative comparison between the electrostatics of an accumulative layer of metal cations and a steady-state concentration of H_3_O^+^ at a charged electrode interface typically requires empirical data incorporated into continuum models spanning the electric double layer.^38^ In this context of more holistic models, ongoing progress in machine learning interatomic potentials with direct inclusion of electrostatic terms (ec-MLIPs),^39–48^ and possibly nuclear quantum effects for H^+^, holds great promise for more accurate simulation of electrochemical interfaces and associated (dynamic) interfacial electrostatic fields. In spite of the limitations of the adopted models and methods, the calculated shift on the CO_2_ LUMO below the Fermi level and the ensuing formation of activated *CO_2_^–^ species for both K^+^ and H_3_O^+^ ions (Figure 5) is qualitatively compelling. We believe the incapability of currently available or emerging ec-MLIPs (as well as classical force fields) to provide direct information (at the accuracy level of DFT) on electronic structure descriptors (e.g., the CO_2_ LUMO) justifies our choice in using DFT for computing atomistic observables not directly accessible by the experimental methods used in the manuscript.

The PDOS of the chemisorbed CO_2_ present in both the K^+^- and H_3_O^+^-containing systems where two cations are present (Figure 5) show similar orbital energies of the now-occupied LUMO (i.e., ca. 2.5 eV below the Fermi level), suggesting that the final adsorbed state is indeed equivalently stable in equivalently charged systems irrespective of cation identity. It is worth noting that these results and conclusions hold for the *CO_2_^–^ intermediate ground state with no information on the actual barrier for electron transfer (e.g., the reaction rate-determining step and associated overpotential in the two systems). Having said that, an estimate of this barrier can be inferred by the calculated LUMO position with respect to the metal Fermi level,^36^ as shown in Figure 4. It should be noted that inclusion of water in the local environment of CO_2_ would mediate the LUMO shifts for a physisorbed linear CO_2_ molecule beyond what is described in Figure 4 and in Notes S3, which do not include water. While the PDOS of chemisorbed *CO_2_^–^ indicates that the LUMO→HOMO energy shift is independent of the local environment, this is not true of the physisorbed CO_2_ along the reaction coordinate to chemisorption.

Our analysis suggests a stronger propensity for electron addition to CO_2_ in the presence of K^+^ in comparison to the H_3_O^+^ system, in line with the larger overpotential required for CO_2_ reduction in the absence of alkali cation reported in this work. Despite this, the calculated CO_2_ LUMO in Figure 4 is lowered in energy by the presence of either cation, relative to that of the CO_2_ LUMO in their absence. While the electronic effects arising from cation identity are influential on the energetics of the chemisorption (a point expanded upon in Note S4), these simulations point out the viability of a CO_2_ reduction mechanism stabilized to a greater or lesser extent by either species.

### Operando Spectroelectrochemistry

We implement a set of spectroelectrochemical testing whereby the potential-dependent response of adsorbed species bound on Au surfaces is demarcated from its nonadsorbed equivalent. Recollecting the work by Gu and co-workers (cf. Introduction section),^19^ it is important to look into the behavior of adsorbed species at potentials around the PZC. However, we have demonstrated that the formation of CO_2_ intermediates without adding metal or organic cations requires either a very negative potential relative to the PZC or an extreme experimental condition that can perturb the interfacial microenvironment.

To enable the operando observation of adsorbed species at potentials close to the PZC (i.e., 0.04 V on Au in 1 mM H_2_SO_4_),^17^ we devise a model system to which CO is introduced as a reporter molecule to study the effect of interfacial microenvironment on the local electric fields (cf. Note S5, Figures S14 and S15, and Methods).^49,50^ Figure 6 shows VSFG spectra obtained with and without metal cations in the model system. For electrolyte purged with CO, a number of resonant peaks are observed in the CO stretch region of the VSFG spectra. In both the presence and absence of K^+^, two prominent bands are observed at 2102 and 2134 cm^–1^ at +0.28 V, assigned to CO linearly atop a singular Au atom (CO_L_) and solvated CO in the ordered region close to the electrode surface, not directly bound to the Au surface (CO_Solv_), respectively. A more detailed discussion of assignments of spectral features is in Note S6 (Figure S16). To guide the eye, two dashed lines are drawn in Figure 6, marking CO_L_ and CO_Solv_.

**Figure 6:**
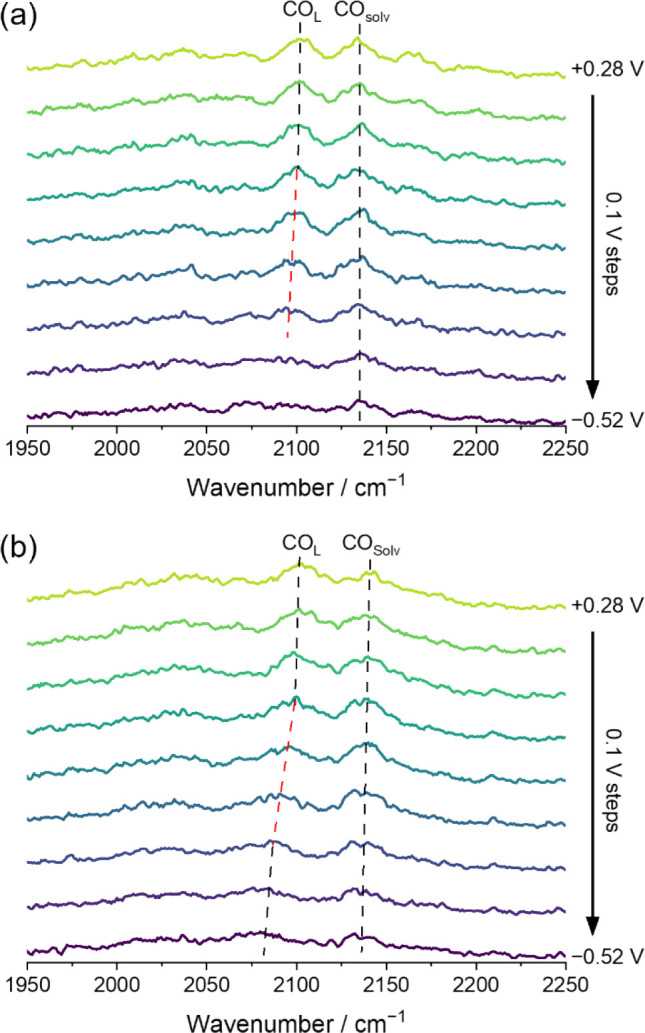
VSFG spectra obtained in CO-purged 0.1 M H_2_SO_4_ solutions in the (a) absence and (b) presence of 0.1 M K_2_SO_4_ using a 42 nm-thick amorphous Au electrode under potential control. The spectra are given at 100 mV intervals while the potentials are reported vs SHE. All spectra were recorded with a 700 fs-nIR delay. CO_L_ and CO_Solv_ bands are indicated by the dashed lines.

The assignment of 2134 cm^–1^ to solvated CO is based on the similarity of this vibrational wavenumber to that of the gas phase and CO solvated by H_2_O.^51^ Consistent with this assignment, the CO_Solv_ peak positions at around 2134 cm^–1^ are mostly unaffected by the applied potential, in line with expectations that these reporter molecules lie farther away from the electrode and thus are exposed to lower electric field strengths. The CO_L_ band has previously been used as a reporter of field strength,^49,52–54^ and we do so here. As this species is chemisorbed to the Au electrode surface, it is in the environment most pertinent to the conversion of CO_2_ to *CO_2_^–^ (*vide supra*).

The key difference observed is that the CO_L_ band position is more greatly shifted between +0.28 and −0.32 V in 0.1 M K_2_SO_4_. For the purposes of understanding the vibrational tuning and changes to the local electric field, the position and intensity of CO_L_ alone is considered. To obtain the quantitative peak center and peak intensity data, the spectra were fitted to a combination of Voigt and Gaussian functions—full details available in Note S6 (Figure S17).

In Figure 7, for each electrolyte, there are three regions that show an approximate linear relationship of CO_L_ vibrational wavenumber with applied potential: (i) + 0.28 to −0.02 V (the latter is around the PZC), (ii) −0.02 to −0.37 V, and (iii) −0.37 to −0.52 V (the latter corresponds to the onset of HER). During HER, the intensity of the CO_L_ band decreases. Vibrational wavenumbers of species at charged interfaces are dependent on both coverage and field experienced.^55^ Hence, we exclude region (iii) from our analysis. Similarly, around the PZC, the local environment of the CO_L_ will be changing as the charged ions within the double layer reorientate; hence, we exclude region (i). During region (ii), the intensity of the CO_L_ band remains approximately constant, and the local environment is expected to remain largely unchanged; hence, we use this potential range to infer relative field strengths between the two electrolyte solutions.

**Figure 7:**
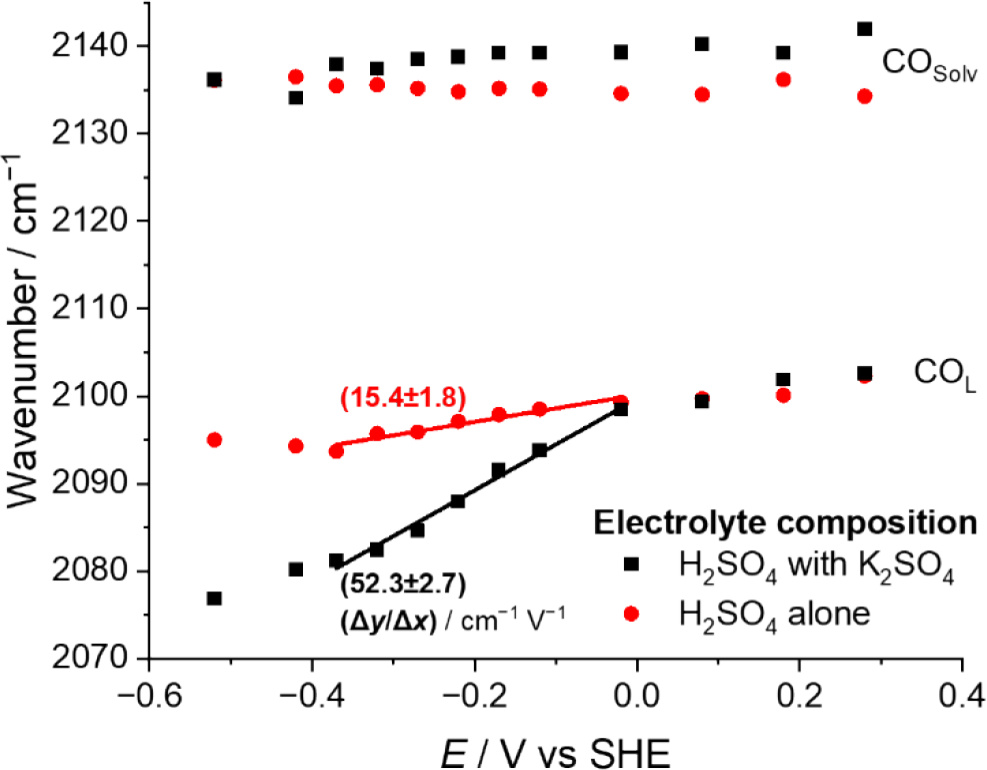
Wavenumbers of the CO_L_ and CO_solv_ band centers across the potential range for (black square) 0.1 M H_2_SO_4_ and 0.1 M K_2_SO_4_ solution and (red circle) 0.1 M H_2_SO_4_ alone solution. The linear fits are given between −0.37 and −0.02 V with the gradients of these fits indicated.

The gradient of the peak position of the CO_L_ mode in region (ii) is 15.4 cm^–1^ V^–1^ without adding metal cations and 52.3 cm^–1^ V^–1^ with the addition, indicating that the electric field strength in the Stern layer is approximately 3.5 times stronger in the presence of K^+^. In other words, even when K^+^ is not added, there is a significant electric field present at the electrode–electrolyte interface, albeit not as large as K^+^. This electric field will have a large effect on the stabilization of the intermediates, with the effect becoming increasingly important as the negative charge of the electrode surface is increased.

## Conclusions

We have shown that the electroreduction of CO_2_ on Au surfaces can occur without adding metal or organic cations. The computational modeling suggested that the transformation of CO_2_ into an adsorbed species *CO_2_^–^ can take place irrespective of cation identity, supporting a mechanism governed by the surface charge density of the electrode. Models (Figure 4) also highlighted that the neutral CO_2_ LUMO is lowered by an electrostatic effect from a metal (K^+^) and nonmetal cation (H_3_O^+^) alike, suggesting that mediation of *CO_2_^–^ intermediate production is possible regardless of the cation identity, which is in excellent agreement with our experimental, real-system observations (Figures 1–3). However, comparison between the results of the simulations of K^+^ and H_3_O^+^ reveals that the former induces more pronounced negative shifts of the CO_2_ LUMO, suggesting that more negative overpotential would be required to initiate the electroreduction in the absence of K^+^. An analogous conclusion can be drawn from operando spectroelectrochemical studies. Here, the behavior of a field reporter molecule chemisorbed to Au surfaces was monitored with or without K^+^ introduced to the system. These experiments demonstrated that although the field at the electrode–electrolyte interface is greater in the presence of K^+^, an appreciable field is observed in the absence of additional K^+^.

On the basis of the foregoing findings, we conclude that the electroreduction of CO_2_ on Au surfaces does not necessarily require the presence of metal or organic cations, but a sufficiently negatively charged interface with respect to the potential of zero charge in the presence of spectating protons, to mediate the transformation of CO_2_ into the intermediate species. We find that this is possible owing to (i) stabilizing electrostatic interaction with H_3_O^+^ within the double layer, (ii) stabilization of intermediates at the electrode surface arising from the interfacial electric field of the charged surface, and (iii) initiation of charge transfer owing to Fermi level of the Au surface becoming higher in energy than the LUMO of *CO_2_^–^ as the Au electrode is increasingly more negatively charged.

## Methods

### Electrolysis Cell Configuration

Essentially, we adopted a cell setup used in our previous work.^17^ To minimize impurities and contaminants, we used a disposable container (Nalgene; polypropylene copolymer, 125 mL, Thermo Scientific) as an electrolysis cell after rigorous rinsing with deionized water (18.2 MΩ cm; Direct-Q 3 UV, Merck), as well as acid cleaning, whenever needed. The cell lid was 3D-printed (UltiMaker^3^) with nylon polymer filament. To ensure airtightness, all cell components were assembled with O-rings.

As the working electrode, a 2 mm-diameter Au disk electrode (CHI101, CH Instruments, Inc.) was used after polishing with four different sizes of diamond suspensions (MetaDi Supreme, Buehler), from 3 μm to 1, 0.25, and 0.05 μm, with rinsing and sonication before and after individual polishing steps.

As the counter electrode, Au wire (99.95%, Advent Research Materials) was used after sanding and rinsing. Only when performing the scavenger experiments was the counter electrode chamber physically separated from the main cell chamber by a porous polytetrafluoroethylene membrane (Omnipore Membrane Filters; 5.0 μm pore size, Merck) and filled with Au scavengers (Phos-4; mercaptoalkyl 1 silica, PhosphonicS; prior to use, it was washed with deionized water); the electrolyte was able to transport between the main cell chamber and counter electrode chamber through the membrane.

As the reference electrode, a double-junction chamber containing Pt wire (013597 and 013375, ALS Co., Ltd.) was filled with 0.5 M H_2_SO_4_ and saturated with H_2_ by conducting electrolysis at −3 V against the Pt auxiliary electrode until the 2 C of charge flowing, and the reference electrode was made freshly whenever required.

As the electrolyte, 1 mM H_2_SO_4_ solution was prepared using the ultrapure H_2_SO_4_ (≥99.999% as a metal basis; Aldrich). Only when performing the chelation experiments was 1 mM of 18-crown-6 ether (≥99.0%, Sigma-Aldrich, China) added to the 1 mM H_2_SO_4_ solution. The level of impurities in the electrolytes was measured using inductively coupled plasma mass spectrometry (NexION 2000, PerkinElmer Inc.). Prior to electroanalysis or bulk electrolysis, the electrolyte was purged using Ar (99.998%, BOC), CO_2_ (99.97%, BOC), or CH_4_/CO_2_ (1% CH_4_ in CO_2_ balance, BOC) at 20 cm^3^ min^–1^ for at least 30 min.

### Electroanalysis

Electroanalytical signals were monitored using a potentiostat (SP-200, BioLogic). The signals were *iR*-corrected by 100%, and the uncompensated resistance was obtained using potentiostatic electrochemical impedance spectroscopy at the OCP. CV was conducted without agitating the electrolyte, scanning from the OCP to an *E*_min_, to +1.1 V vs SHE, and then back to the OCP at 50 mV s^–1^. The following *E*_min_ values were chosen: −1.4, −1.8, −2.2, −2.6, −3.0, −3.4 V. For modified condition experiments (e.g., with scavenger or chelating agent), −3.4 V was used as *E*_min_.

### Bulk Electrolysis

Bulk electrolysis was carried out in CO_2_-saturated 1 mM H_2_SO_4_ solution on a bare Au electrode in the presence of CH_4_ internal standard (1%) using chronoamperometry at −1.4, −1.8, −2.2, −2.6, −3.0, or −3.4 V. Under the carrier gas of He (99.9999%, BOC), gas chromatography (6890N G1530A, Agilent) was performed to measure the gas products, as well as the concentration of the internal standard. The obtained chromatograms were calibrated using a commercial calibration gas mixture (2.8% H_2_, 3500 ppm of CH_4_, 2.8% CO, etc. in CO_2_ balance; CK Isotopes). Electrolysis was conducted for at least 1800 s with magnetic stirring (stirrer bar: 20 mm long and 7 mm diameter) at 600 rpm (stirrer plate: Guardian 5000, OHAUS). Care was taken to maintain the relative distance and position between the center of the magnetic stirrer bar and the Au electrode as similar as possible across experiments. We noted that their relative location was one of the factors that affected Faradaic efficiencies.

### Simulation Parameters

All DFT calculations were performed in the Projector-Augmented Wave (PAW) approach with the VASP 6.3 code using a planewave basis set with periodic boundary conditions.^56,57^ The planewave energy cutoff was set to 500 eV. The PBE approximation to the exchange–correlation functional was used alongside Grimme’s D3 van der Waals corrections with Becke–Johnson damping.^58,59^ The basis 1 × 1 simulation supercell was orthorhombic, of dimensions (8.702 Å × 10.048 Å × 34.737 Å). For 2 × 1 supercells, the cell was doubled in the *x*-dimension. Calculations incorporated a three-layer Au(111) surface slab. The bulk Au lattice parameter used for slab construction was previously converged in VASP with respect to interatomic distance and *k*-point sampling to *a* = 4.102 Å. For the water-layer calculations in the 2 × 1 supercells, the bottom layer atomic positions were kept fixed. VASP’s native dipole correction scheme^60,61^ was applied normal to the slab to cancel spurious dipole induction between cell images. Gaussian partial occupancy smearing of σ = 0.2 eV was used. All systems containing water layers were converged to forces within 0.1 eV Å^–1^ per atom (self-consistent field (SCF) calculation tolerance = 10^–6^ eV). All systems containing direct adsorption of bare ionic species onto Au(111) were converged to forces within 0.03 eV Å^–1^ per atom using Gamma-centered *k*-point sampling of (4 × 3 × 1). Low-tolerance molecular dynamics runs in the NVT ensemble were used to obtain a pre-relaxation geometry for the water-containing systems (SCF tolerance = 10^–4^ eV, Γ-point only, using a Nosé–Hoover thermostat with the “SMASS” parameter set to obtain a thermostat oscillation period of approximately 80 fs).^62^ Runs were originally carried out for approximately 2.5 ps for a Cs-containing system to obtain a thermodynamically stationary system, and a further 0.5 ps run was performed upon substituting K and H_3_O as the charge-donating species for each system. To obtain PDOS data with a Fermi level consistent with the bare adsorption calculations, all snapshots of systems containing water layers were coarsely relaxed to within forces of 0.1 eV Å^–1^ per atom (SCF tolerance = 10^–6^ eV) at a reduced (2 × 3 × 1) *k*-point scheme to account for the increase in the cell size in the *x*-direction. PDOS plots were created from VASP PROCAR output files written with 3000 data points using scripts from VASPKIT.^63^ Charge partition and analysis was performed using the Bader method.^64^ All geometry construction was performed using the Atomic Simulation Environment libraries,^65^ with coarse initial relaxation of the water layers performed using the extended tight binding Hamiltonian^66^ to obtain input geometries for VASP.

### VSFG Cell Design

The cell consists of three pieces of polyether ether ketone (PEEK), two window holding pieces and one central piece, which holds the electrolyte and the electrodes (Figure S14). Between each window and each piece of PEEK was an *N*-butyl rubber (NBR) O-ring to make the seal, protect the windows and make the cell compressible. Two pieces of 10 μm-thick Au foil (99.9%, Goodfellow) were placed in contact with the thin Au working electrode and compressed underneath the NBR O-ring to connect the working electrode to the outside of the cell. Once the cell was assembled, the connection to the working electrode was tested by measuring the resistance between the two pieces of Au foil. One of the pieces of Au foil was wrapped around the exposed end of a Kynar-insulated Ag-plated Cu wire (RS Components). The wire was taped to the cell at each side of its connection with the Au foil to secure the connection and limit the tension on the foil.

The counter electrode was a 500 μm-diameter bare Au wire (99.95%, Advent Research Materials) and was polished with P1200 SiC sandpaper before being rinsed with deionized water (18.2 MΩ cm; Direct-Q 3 UV, Merck). The counter electrode was introduced through to the cell, through a septum in the top of the cell, so that ca. 30 mm of the wire was inside the cell, and ca. 5 mm of the wire was placed inside the main chamber of the cell. The wire is held at ca. 5 mm from the working electrode and ca. 5.5 mm from the reference electrode. The reference electrode was a 2 mm-outer-diameter leak-free Ag/AgCl reference electrode (Innovative Instruments Inc.). The reference electrode introduced to the side of the cell through a septum so that the working area was ca. 8 mm into the main chamber of the cell but still away from any transmitting light from the laser beams. The working area of the reference electrode was ca. 5 mm from the working electrode.

### VSFG Au Electrodes

The substrates for the electrodes were 2 mm-thick, 40 mm-diameter CaF_2_ polished windows (IR-grade, Crystran). Prior to deposition, the windows were prepared by stepwise sonication in acetone, isopropyl alcohol, deionized water, and then deionized water again. Windows used on the opposing side of the cell were also prepared in the same manner. Au was deposited onto the substrates by thermal evaporation under vacuum at between 7.5 × 10^–6^ and 2.5 × 10^–5^ mBar. The electrodes were grown at a rate of 0.1 ± 0.05 Å s^–1^ to a thickness of approximately 42 nm. The growth rate and thicknesses are determined by an in situ quartz crystal microbalance.

### VSFG Experiments

All electrochemical measurements conducted prior to VSFG measurements were conducted using a potentiostat (SP-200, BioLogic). The potential of the reference electrode was standardized against two separate systems: a ferrocene/ferrocenium couple and a master reference electrode. Once the cell was assembled and filled with 0.1 M H_2_SO_4_ (≥99.999% as metal-basis, Aldrich, USA). The cell was purged with either Ar (99.998%, BOC) or CO (100%–CP grade, BOC) at 10 mL min^–1^ for 10 min.

To generate the nIR pulse, 10% (1 W) of the output of PHAROS-PH1-SP (Light Conversion, 1030 nm, 10 kHz, 10 W, 170 fs pulse duration) is passed through an etalon (SLS Optics) and a delay stage (Thorlabs, LTS300C) to produce a delay-controlled, narrow-band time asymmetric nIR pulse (1030 nm, 10 kHz, ∼1.5 ps, ∼13 cm^–1^ line width). This nIR was passed through a half-wave plate (Thorlabs, WPH10M-1030) and polarizer (Thorlabs, LPVIS050-MP2) to obtain horizontally polarized (p-polarized with respect to the sample reflection) light. The nIR light was focused using a 25 cm lens (Thorlabs, LB1056) and transmitted through a 950 nm-long pass filter (Thorlabs, FEL0950) to filter out second harmonic generated 515 nm light. The nIR is brought in through the back of the CaF_2_ and thin Au working electrode at an incident angle ∼45°, where the nIR beam was measured as ∼20 mW with a spot size of ∼150 μm (fwhm).

To generate the mIR pulse, 80% (8 W) of the PHAROS-PH1-SP output was directed into an IR OPA (Light Conversion, Orpheus-One-HE) to generate a tunable broadband IR beam at the chosen frequency (10 kHz, 170 fs pulse duration and ∼130 cm^–1^ at 2050 cm^–1^) with horizontal polarization (p-polarized with respect to the sample reflection). Attenuation of the mIR light from atmospheric H_2_O and CO_2_ was reduced by passing purge-generated gas through the beam path. The beam is focused onto the back of CaF_2_ and thin Au working electrode using an Au parabolic mirror (Thorlabs, MPD249H-M01), where the mIR beam was measured as ∼12 mW with a spot size of ∼350 μm. The resulting SFG beam is passed through a polarizer to selectively sample the horizontally polarized light (p-polarized), before being focused through 150 μm slits and into the spectrograph (Andor, Kymera) before being detected on a CCD camera (Andor, iDus416). For all measured constant potential spectra, background-corrected VSFG signals were accumulated for 120 s at the CCD, at different nIR time delays and applied potentials as specified.

All electrochemical data and potentiostatic control during the VSFG measurements were achieved using another potentiostat (EmStat4S, PalmSens). Once the VSFG signal was optimized, four cycles of CV were conducted, starting from the OCP, going to −0.6 V and then +0.4 V vs Ag/AgCl (leak-free electrode) at 10 mV s^–1^. During potential-controlled spectroscopic measurements, the cell was held at a potential of interest for up to 20 min at a time and the chronoamperometry was performed while background-corrected VSFG signals were accumulated.

### Pre-VSFG Electrochemical Tests

To compare to the ferrocene couple, four cycles of CV were conducted from OCP to +1 V and to −1 V vs Ag/AgCl (leak-free electrode) at 50 mV s^–1^ in the ferrocene-containing solution. A glass carbon disk electrode was used as the working electrode, a flame-annealed Pt wire (99.95%, Advent Research Materials) as the counter electrode, and the leak-free Ag/AgCl electrode as the reference electrode. The solution contained 5 mM of the ferrocene couple (98%, Aldrich, China) and 10 mM of KCl (99%, Thermo Scientific, Germany) in water and was refreshed after every two experiments.

The master electrode measurements were conducted in a two-electrode setup, where the Ag/AgCl leak-free electrode potential was measured at open circuit for 2 min against a master Ag/AgCl reference electrode (in 3 M NaCl; MF-2052, BASi) in the same electrolyte, which was to be used in the corresponding VSFG experiment (i.e., 0.1 M H_2_SO_4_).

## Supplementary Material



## Notes

Notations for adsorbed CO_2_; effect of the applied potential relative to the potential of zero charge; disproving the possibility regarding autogenous cations; decomposing the Au–CO_2_ and ion–CO_2_ interactions; background of spectroelectrochemical measurements; peak assignments for vibrational spectra; cyclic voltammograms obtained on the Au electrode in CO_2_-saturated 1 mM H_2_SO_4_ solution; graph showing the average and standard deviation of the results from 10 individual cyclic voltammograms obtained on Au electrode in CO_2_-saturated 1 mM H_2_SO_4_ solution; cyclic voltammograms obtained on the Au electrode in CO_2_-saturated post-electrolysis 1 mM H_2_SO_4_ solution; gas chromatograms obtained before and after electrolysis from the headspace of the cell in the presence of 1% CH_4_ as an internal standard; PDOS about the Fermi level of CO_2_ physisorbed on Au(111) in the presence of H_3_O^+^ and K^+^; change in LUMO of CO_2_ with the spatial retraction of K^+^; full data set of geometry-relaxed configuration for K^+^- and H_3_O^+^-co-adsorbed systems; side cross section of the VSFG cell and front view of the Au-coated window in the VSFG cell; peak-normalized VSFG spectra at −220 mV vs SHE at different nIR delay times for CO-purged 0.1 M H_2_SO_4_ and 0.1 M H_2_SO_4_ and 0.1 M K_2_SO_4_ solutions; VSFG spectra at different 800 fs nIR delay for Ar-purged 0.1 M H_2_SO_4_ and 0.1 M H_2_SO_4_ and 0.1 M K_2_SO_4_ solutions at different applied potentials vs SHE; smoothed VSFG spectra and their respective fits for CO-purged 0.1 M H_2_SO_4_ and 0.1 M H_2_SO_4_ and 0.1 M K_2_SO_4_ solutions; inductively coupled plasma mass spectrometry analysis of electrolytes; and calculated DFT energies for the configurations labeled in Figure S13 (PDF)
