# Supplementary material for: Carbon Dioxide Electroreduction on Gold without Metal or Organic Cations
Source: ACS Catal. 2025 Jun 18;15(13):11452–62. doi: 10.1021/acscatal.5c02785 (PMC12235593; doi:10.1021/acscatal.5c02785)
Supplement: Supplementary file 1 [file cs5c02785_si_001.pdf]

Supporting Information

Carbon dioxide electroreduction on gold without metal or organic cations

Hansaem Jang,[†] Ciarán O'Brien,[†,‡,‖] Nathaniel J. D. Hill,[†] Adrian M. Gardner,[†,§] Ivan Scivetti,[¶] Gilberto Teobaldi,[‡] and Alexander J. Cowan*,[†]

[†]Stephenson Institute for Renewable Energy (SIRE) and the Department of Chemistry, University of Liverpool, Liverpool L69 7ZF, United Kingdom

[‡]Scientific Computing Department, Rutherford Appleton Laboratory, STFC UKRI, Harwell Campus, Didcot OX11 0QX, United Kingdom

[‖]Central Laser Facility, Research Complex at Harwell, STFC-Rutherford Appleton Laboratory, Harwell Science and Innovation Campus, Didcot OX11 0QX, United Kingdom

[§]Early Career Laser Laboratory and Surface Science Research Centre, University of Liverpool, Liverpool L69 3BX, United Kingdom

[¶]Scientific Computing Department, Daresbury Laboratory, STFC UKRI, Daresbury, Warrington WA4 4AD, United Kingdom

* acowan@liverpool.ac.uk

Table of Contents

Supplementary notes (Note S1–S6)

Supplementary figures (Figure S1–S17)

Supplementary tables (Table S1 and S2)

Supplementary references (ref. S1–S32)

Supplementary notes

Note S1. Notations for adsorbed CO_2

On the notation for chemisorbed CO_2 (in the main text, denoted $*CO_2^-$), previous literature denotes chemisorbed (bent) CO_2 as $*CO_2$ (or CO_2*).[1-3] We have chosen to include a negative charge superscript to reflect the excess charge localized on the $*CO_2$, which is a necessary consequence of the excess electronic density required on the slab model to initiate chemisorption in our computational models. Formally, as the CO_2 undergoes reductive rehybridization with the Au surface, it is true that this chemisorption is a bonding much more than a charging, and the excess charges reported are true only in the formalism of the Bader charge partition of the DFT-calculated charge density. However, given the central importance of the surface charge density to the hypothesis of this reaction mechanism, we have elected to refer to chemisorbed CO_2 as $*CO_2^-$ in order to highlight the localization of this excess surface charge on the CO_2 upon chemisorption.

Note S2. Effect of the applied potential relative to the potential of zero charge

On the basis of the oxygen reduction reaction study, [4, 5] we surmise that the observation by Gu and coworkers[6] is contributed by the relative population of cationic proton donors versus CO_2 at the cathode surface. For example, if the applied potential is located positive of the potential of zero charge (PZC) while negative of the CO_2RR onset potential, cationic proton donors will not be electrostatically attracted to the negatively charged electrode through migration, and both cationic proton donors and CO_2 will arrive at the electrode surface by diffusion or convection; therefore, the likelihood of the hydrogen evolution reaction (HER) to occur by cationic proton donors will be relatively less than a case where the applied potential is negative of the PZC, which in turn could facilitate the occurrence of CO_2RR.

Note S3. Disproving the possibility regarding autogenous cations

Suppose that the Au ions were indeed autogenously generated at the concentration lower than the detection limit but still capable of activating CO_2RR. If this is the case, the repeated use of the same electrolyte should enable CO_2RR at potentials where the electroreduction of CO_2 typically occurs as there exist the residual Au ions in the electrolyte. However, this possibility is disproved by a series of experiments involving a preceding electrolysis at −3.4 V vs SHE (hereinafter all potentials are referenced against SHE unless otherwise specified) and a following electrolysis at −1.4 V, where CO stripping is not observable in the following electrolysis (Figure S9).

Note S4. Decomposing the Au–CO_2 and ion–CO_2 interactions

An attempt is made in the following tests to disentangle the factors contributing to the role of the cation in the downshift in the CO_2 lowest unoccupied molecular orbital (LUMO). Whether it is a purely electrostatic interaction or has relevant contribution from orbital hybridization between the CO_2 LUMO and the cation is investigated with altered configurations of our models given in Figure 4. These are subject to the limitations of GGA–DFT; most notably, an unphysical electronic density falloff with distance. [7]

The change in LUMO energy upon spatially separating (i) the cation and (ii) the Au from the (physisorbed) linear CO_2 molecule is plotted and compared for systems containing (a) H_3O^+ and (b) K^+ as the cationic co-adsorbate in Figure S11. The relative position for both cations to the CO_2 was taken from the position of the proximal ion to the $^*CO_2^-$ in the water layer systems (Figure 5). Before the geometry was further modified. For both systems, the relevant component of the system was retracted 4 Å from the CO_2 along the *z*-axis. As shown, for both systems the separation of the Au results in a lowering of the LUMO energy (at an overall cost to the total calculated energy of the system). Comparing the H_3O^+ and the K^+ separation from the CO_2, the distance-dependent influence of the cation is markedly different.

Further investigations into the decay of the cation interactions on the CO_2 LUMO in these systems are plotted in Figure S12. The effect of the H_3O^+ decays to a lesser extent with distance than that of the K^+ but induces a lower overall energy shift and less broadening of the CO_2 LUMO. For K^+, the LUMO exhibits a non-linearity in energy shift with distance when at short range from the CO_2 as well as significant broadening of the projected density of states (PDOS) peak (Figure S12a), indicative of a degree of orbital hybridization. Taking the same relative positions of CO_2 and cation but swapping K^+ for H_3O^+, it is shown that the rate of change of LUMO energy with distance exhibits a similar curvature but to a far less extent, and with significantly less broadening (Figure S12b). When instead the counterpart proximal H_3O^+ position relative to $^*CO_2$ is used (from Figure 5) as a starting point for the same test, the shift in LUMO is far less pronounced, and approximately linear (Figure S12c), suggestive that the interaction is largely electrostatic in nature.

These models were constructed as follows: the relative position of the proximal K^+ ion to the $^*CO_2$ was taken from the water layer geometry in Figure 5, and transposed into a reference cell containing linear CO_2 physisorbed on bare Au(111) (geometry giving the purple trace of the PDOS in Figure 4). The physisorbed CO_2 was also rotated to align its principal axis with the $^*CO_2$ to maintain its relative orientation to the K^+. The PDOS of the CO_2 was tracked with the incremental removal of the K^+ vertically along the *z*-axis, away from the CO_2 and Au(111). To generate the data in Figure S12b, the geometry of the proximal H_3O^+ in the water layer model (Figure 5) was used to replace the K^+ ion. These two plots are also compared to the change in

LUMO energy when the original relative position of the water layer H_3O^+ to the $*CO_2^-$ was retracted by an incremental vertical shift (Figure S12c). In contrast to the K^+–water layer system, the H_3O^+ was positioned laterally from the CO_2, rather than above it. The sets of sample configurations of the K^+ and H_3O^+ are available in Figure S13 (cf. Table S2).

Note S5. Background of spectroelectrochemical measurements

Surface-sensitive vibrational spectroscopy can be particularly useful for understanding the chemical and physical structuring at electrochemical interfaces, by observing the number and position of different vibrational modes. [8, 9] The frequencies of these vibrational modes are sensitive to changes in the local environment such as bonding environment, intermolecular environment and the local electric field. These changes in environment can be induced by changing the potential applied to an electrode, either directly by changing the local potential and electric field, or indirectly by changing the chemical structure and arrangement of molecules within the electrical double layer. As the potential applied to an electrode is changes, these changes can be observed in the vibrational spectra as tuning of the vibrational frequencies. If it can be assumed that over given potential range, the chemical structure and environment for a specific chemical species in unchanged, then the tuning observed in the vibrational spectra can be directly related to the changes in local electric field. [10-14]

Carbon monoxide is commonly used in vibrational spectroscopy as a reporter molecule to study the tuning rates and inform on changes to the local electric field at CO_2RR electrode–electrolyte interfaces. [14-19] The frequency and intensities of interfacial CO stretch vibrations can be followed to understand structure and field changes across an applied potential range. However, the observation of interfacial species in electrochemical cells using vibrational spectroscopy can be challenging due to low relative concentrations of interfacial species, making it difficult to separate the signals from interfacial and bulk species. Surface-sensitive techniques are, therefore, key to understanding the structure and properties of the interfacial region under electrochemical conditions. [8, 9, 20] Vibrational sum frequency generation (VSFG) has been used to study many of these electrochemical systems due to its inherent surface sensitivity. For the measurements discussed here, a tuneable broadband mid-infrared (mIR) and a 1028-nm (i.e. near-infrared, nIR) time-asymmetric narrowband beam were overlapped at the interface of interest to produce field oscillating at the sum of the frequencies of the incident fields (Figure S14). At the interface, the incident oscillating electric fields can interact with vibrational transitions of interfacial species which are resonant with the mIR frequency to produce resonant SFG signal. These fields can also interact with the electrons of the interface to produce non-resonant (largely frequency-independent) SFG signal. The resonant and non-resonant components can constructively or

destructively interfere with each other, resulting in a complex line shape. By introducing a time delay between the mIR pulse and the nIR pulse, the non-resonant signal can be suppressed, allowing the resonant spectrum to be more easily interpreted. [21] However, if the time delay is too large, resonant information is lost. It was found that for these measurements, a delay of approximately 700 fs was the best compromise between observance of resonant signal and non-resonant suppression (Figure S15). Further details of VSFG and non-resonant suppression in VSFG can be found elsewhere; [8, 22] and details of the VSFG experimental apparatus used for these measurements have been reported previously, [16, 23] and detailed below.

While VSFG is a powerful tool for understanding electrode–electrolyte interfaces, coupling to electrochemical cells requires careful consideration. Approaching the interface through an aqueous electrolyte leads to mIR losses owing to large infrared (IR) absorption. This arrangement becomes feasible if the aqueous layer is reduced to an ultrathin layer where the absorption of IR radiation is low. [16, 23] However, under these conditions, mass transport mechanisms and slow dissipation of evolved gas becomes significant. Most conductive electrode materials of interest are non-transparent, which makes bringing the beams through the electrode medium also difficult. Electrodes can be reduced to just a few nanometers to allow sufficient propagation of the oscillating electric field at the expense of stability under representative current densities due to resistive degradation. Recently, Baker and coworkers have employed Au electrodes on the order of 10s of nanometers to enable so-called 'backside VSFG' measurements, in which the mIR, nIR and VSFG radiation propagates through the ultrathin electrode. In those experiments, potential-dependent vibrational wavenumbers of CO at the Au interface was used as an indicator of local field strength. [14, 15, 18, 19, 24-26] In this arrangement, VSFG signal is achieved, without compromised mass transport geometry, while maintaining relative electrode stability at greater than 1 mA cm^{-2}. In this work, we used this experimental design to observe CO in K^+ and K^+-free environments to understand the relative tuning rates under potential control, the relative magnitude of the local electric fields and the relative structuring of the Stern and diffuse layers of these systems.

Note S6. Peak assignments for vibrational spectra

In the absence of K^+, two prominent bands are seen at 2102 and 2134 cm^{-1}, respectively, at +0.28 V (Figure 6a). When increasingly negative potential is applied to the electrode, the band at 2102 cm^{-1} shifts to lower wavenumbers, broadens, and becomes weaker in intensity. Here, we assign this peak as CO bound linearly to a singular Au atom (CO_L) in accordance with the past VSFG and surface-enhanced infrared absorption spectroscopy studies on Au surfaces which observed bands in the 2100–2120 cm^{-1} range assigned to this species. [18, 27-30]

The band at 2134 cm^{-1} at +0.28 V, by contrast to CO_L, does not show a significant potential dependent wavenumber. The intensity also remains roughly constant between +0.28 and −0.32 V and decreases at more negative potentials around the onset of HER. Baker and coworkers observe a weak band at a similar frequency on similar Au electrodes. [18] They assign this peak to multiple linear binding sites due to the observed tuning and adoption behavior. Density functional theory-supported IR spectroscopy of gas phase micro-solvation clusters has shown the stretching frequency of CO solvated by 4 water molecules as 2144 cm^{-1}. [31] The observance that the 2134 cm^{-1} band does not shift significantly with potential is consistent with that the CO resides farther away from the electrode and thus are exposed to lower electric field strengths. However, since a VSFG signal is observed, which requires overall net ordering, we assign this band to solvated CO which is not chemisorbed to the Au electrode surface, in the ordered region close to the electrode surface and denote this as CO_{Solv}.

Other bands are observed, which can be assigned to multiple CO sites with similar vibrational wavenumbers, confirmed through performing experiments in Ar purged solutions (Figure S16). For instance, we observe a feature at 2036 cm^{-1} (Figure 6a), which has previously been assigned as a CO linearly bound to steps and terrace sites which have similar vibrational wavenumbers. [32] This band changes in shape and linewidth, which we attribute to changes in relative population of CO bound at these sites with applied potential. Deconvolution of this band is challenging with the given signal-to-noise, as such we assign this band collectively as CO bound at multiple undercoordinated linear sites (CO_{ML}). A weak band at 2076 cm^{-1} (−0.22 V) is most clearly observed at most positive and most negative potentials in H_2SO_4 solutions, not present in Ar-purged solutions; hence assigned to CO@Au. A further weak band is observed at 2164 cm^{-1}, which is constant in wavenumber with applied potential and decreases in intensity at more negative potentials. This band is present in CO-purged 0.1 M H_2SO_4 and is not clearly observed when K^+ is introduced or when the cell is purged with Ar (Figure S16). Typically, CO frequencies decrease when bound to metal surfaces, and as noted, this band is higher in wavenumber than solvated CO and does not show a potential dependence on vibrational wavenumber; hence we conclude that this band arises from CO within the electrochemical double layer and is sensitive to the nature of the species present.

In the presence of K^+, the band at 2076 cm^{-1} is not clearly resolved from the CO_L band and the 2134 cm^{-1} band is not observed, while 3 main features remain (Figure 6b), once again assigned to CO_{Solv} (2142 cm^{-1}), CO_L (2103 cm^{-1}) and CO_{ML} (2029 cm^{-1}).

To fit the spectra, we have used a combination of Gaussian and Voigt functions: Gaussian curves to model the residual non-resonant contribution and Voigt curves to model resonant peaks. It must be noted that there is no single solution to fit these spectra, and the fitting is limited by pixel resolution, smoothing and signal-to-noise and the number of Voigt functions chosen justified from the description in Note S6. It

was found that for the K^+-free spectrum, 2 non-resonant Gaussians and 5 resonant Voigts achieved the best fit (Figure S17a), while for the electrolyte with K^+, 2 non-resonant Gaussians and 5 resonant Voigts provided the best fits (Figure S17b). Due to the assignment of multiple sites in the 2010–2075 cm^{-1} range, it is challenging to fit all these features effectively. While it is noted that the fitting around CO_{ML} is not optimal, the changes in population of these sites would make them poorly suited for use as reporter modes. In addition, overfitting these modes could affect the quality of fit around CO_L, the reporter mode in this study.

Supplementary figures

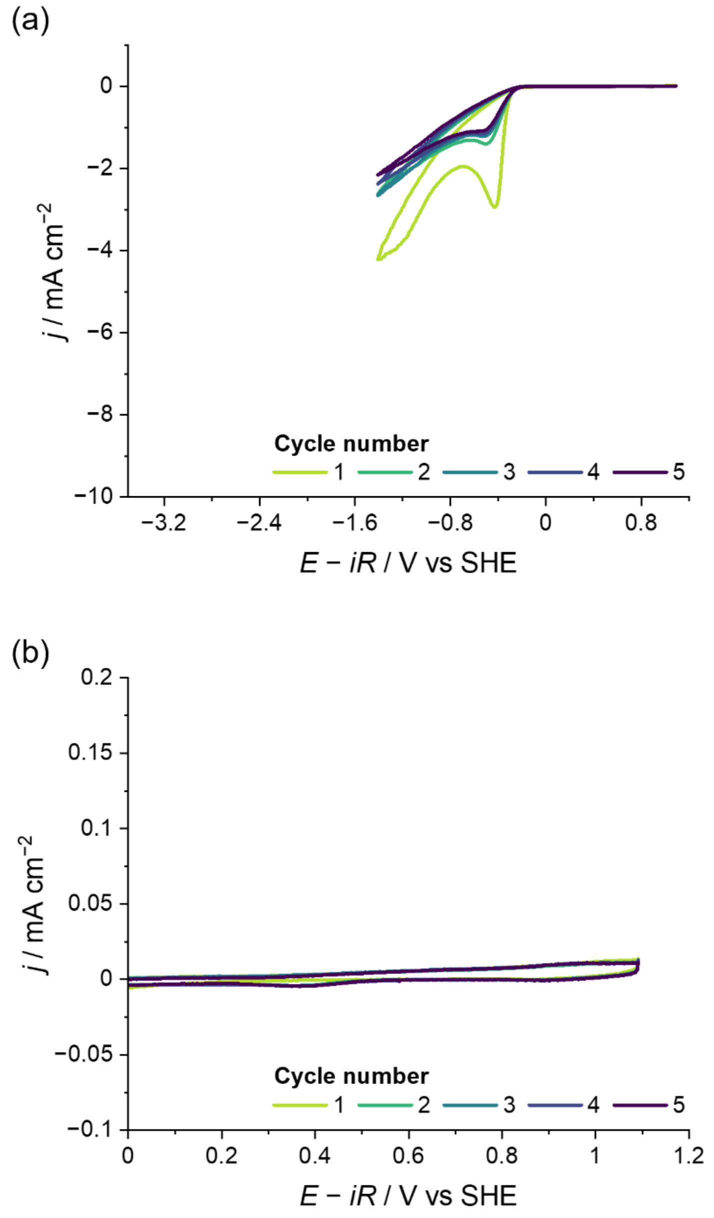

Figure S1. Cyclic voltammograms obtained on Au electrode in CO_2-saturated 1 mM H_2SO_4 solution: (a) Full potential window, (b) Narrower potential window with a magnified *y*-axis scale. The electrode potential was initially scanned from the OCP to −1.4 V, then to +1.1 V, and back to the OCP at 50 mV s^{-1}.

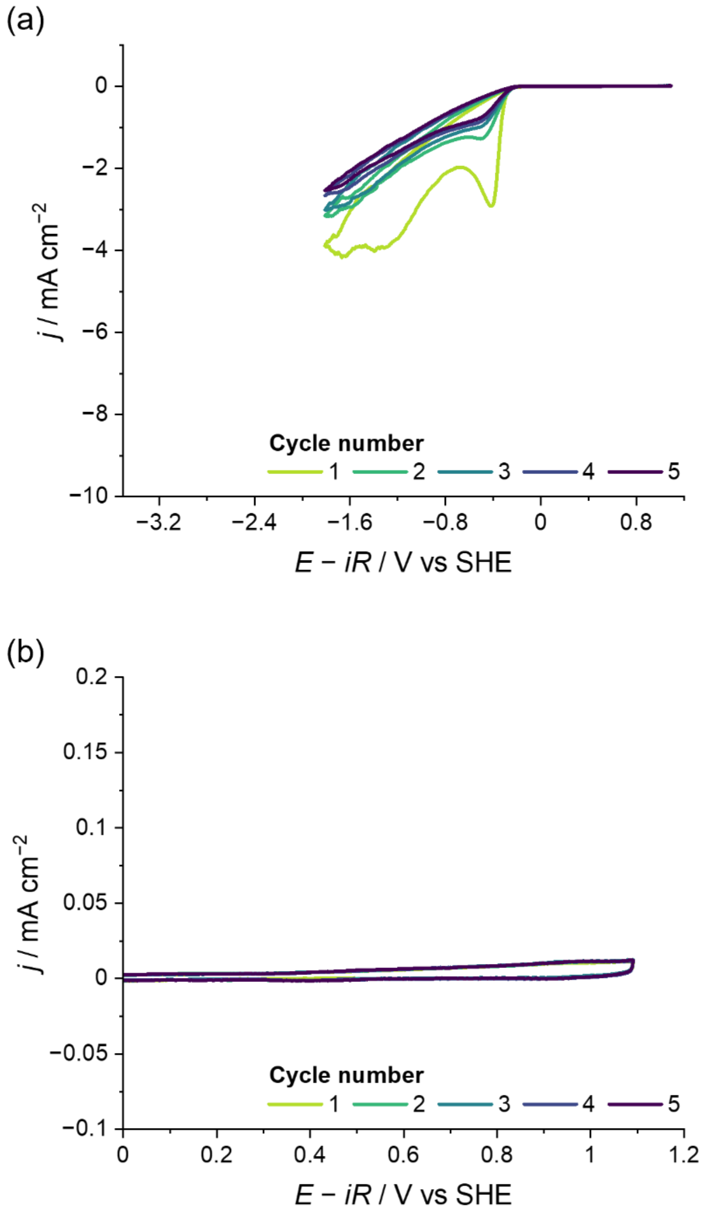

Figure S2. Cyclic voltammograms obtained on Au electrode in CO_2-saturated 1 mM H_2SO_4 solution: (a) Full potential window, (b) Narrower potential window with a magnified *y*-axis scale. The electrode potential was initially scanned from the OCP to −1.8 V, then to +1.1 V, and back to the OCP at 50 mV s^{-1}.

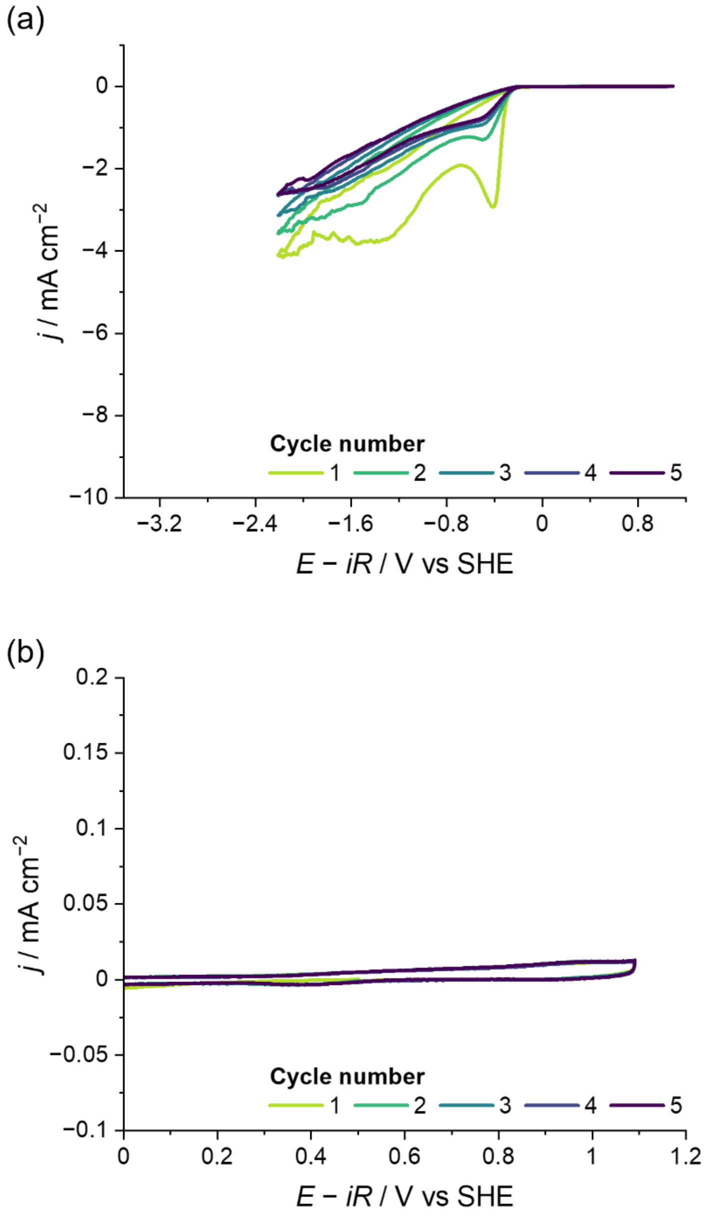

Figure S3. Cyclic voltammograms obtained on Au electrode in CO_2-saturated 1 mM H_2SO_4 solution: (a) Full potential window, (b) Narrower potential window with a magnified *y*-axis scale. The electrode potential was initially scanned from the OCP to −2.2 V, then to +1.1 V, and back to the OCP at 50 mV s^{-1}.

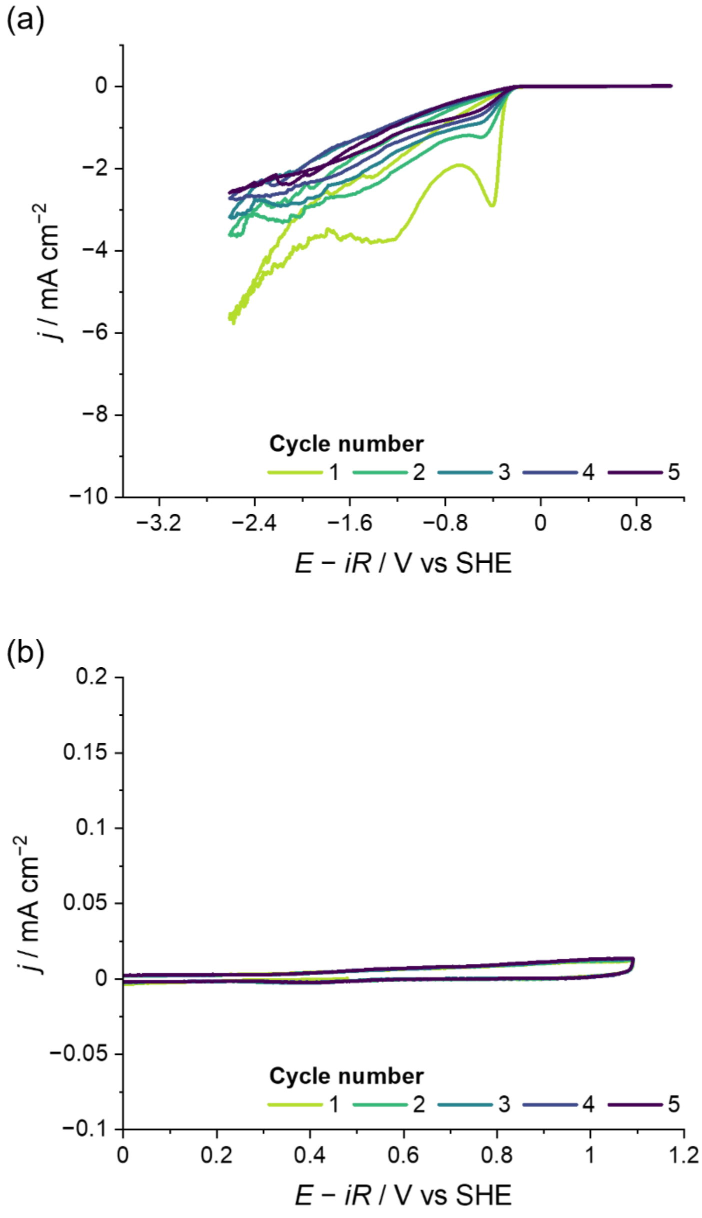

Figure S4. Cyclic voltammograms obtained on Au electrode in CO_2-saturated 1 mM H_2SO_4 solution: (a) Full potential window, (b) Narrower potential window with a magnified *y*-axis scale. The electrode potential was initially scanned from the OCP to −2.6 V, then to +1.1 V, and back to the OCP at 50 mV s^{-1}.

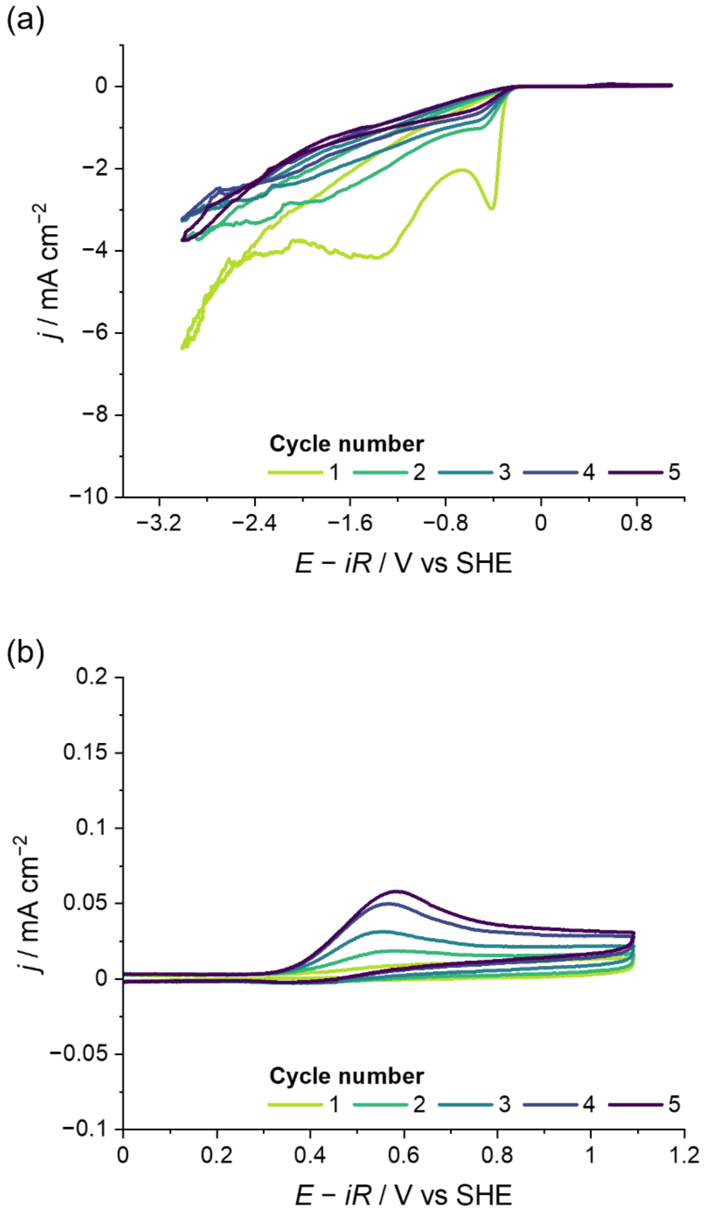

Figure S5. Cyclic voltammograms obtained on Au electrode in CO_2-saturated 1 mM H_2SO_4 solution: (a) Full potential window, (b) Narrower potential window with a magnified *y*-axis scale. The electrode potential was initially scanned from the OCP to −3.0 V, then to +1.1 V, and back to the OCP at 50 mV s^{-1}.

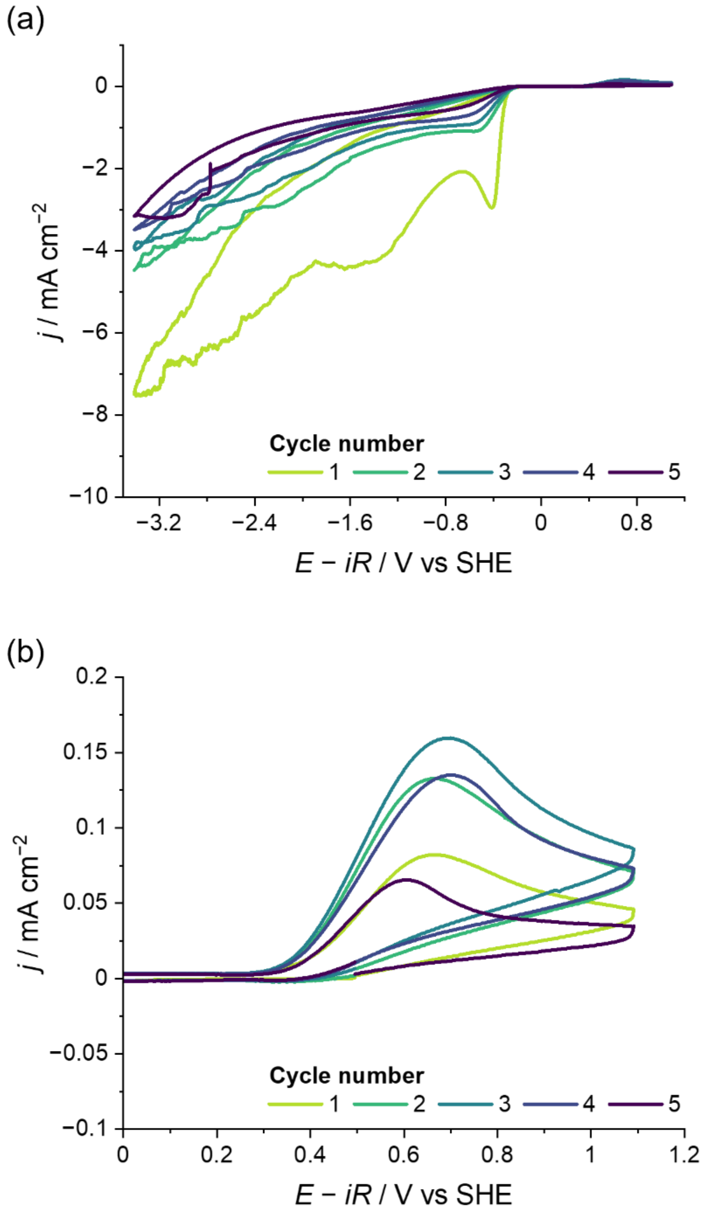

Figure S6. Cyclic voltammograms obtained on Au electrode in CO_2-saturated 1 mM H_2SO_4 solution: (a) Full potential window, (b) Narrower potential window with a magnified *y*-axis scale. The electrode potential was initially scanned from the OCP to −3.4 V, then to +1.1 V, and back to the OCP at 50 mV s^{-1}.

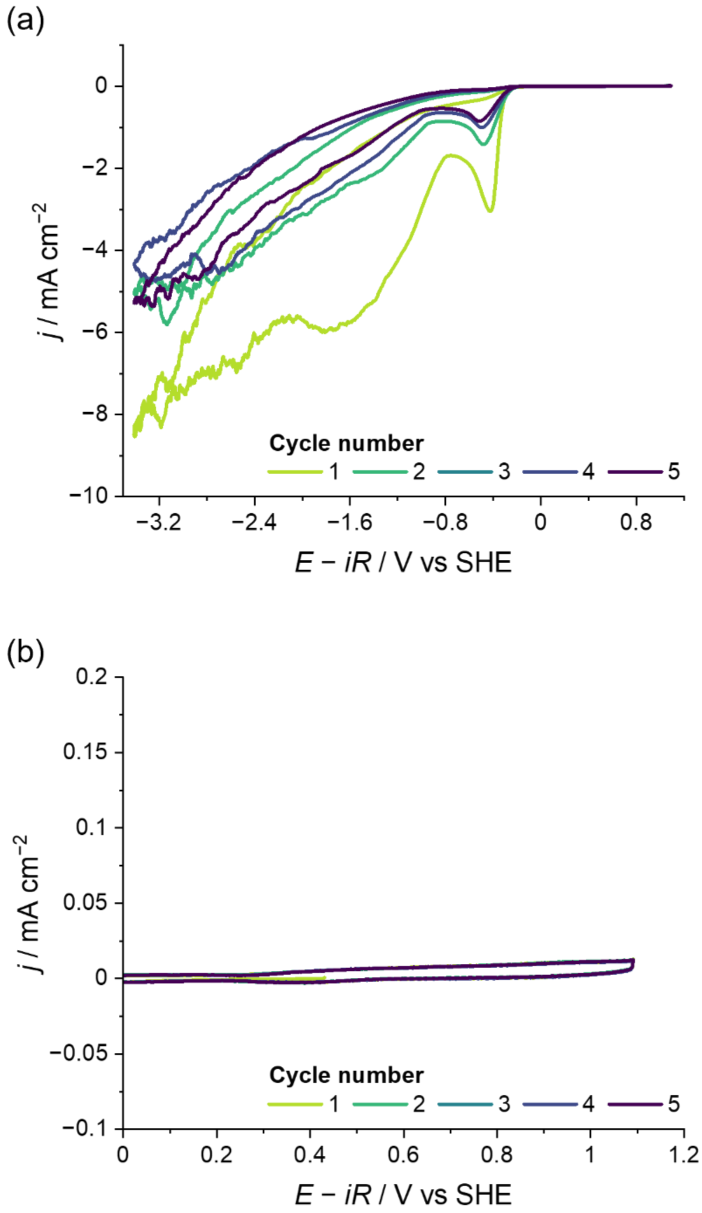

Figure S7. Cyclic voltammograms obtained on Au electrode in Ar-saturated 1 mM H_2SO_4 solution: (a) Full potential window, (b) Narrower potential window with a magnified *y*-axis scale. The electrode potential was initially scanned from the OCP to −3.4 V, then to +1.1 V, and back to the OCP at 50 mV s^{-1}. Comparison between this curve and a curve obtained in CO_2 (cf. Figure 2) confirms that CO can be produced only in the presence of CO_2.

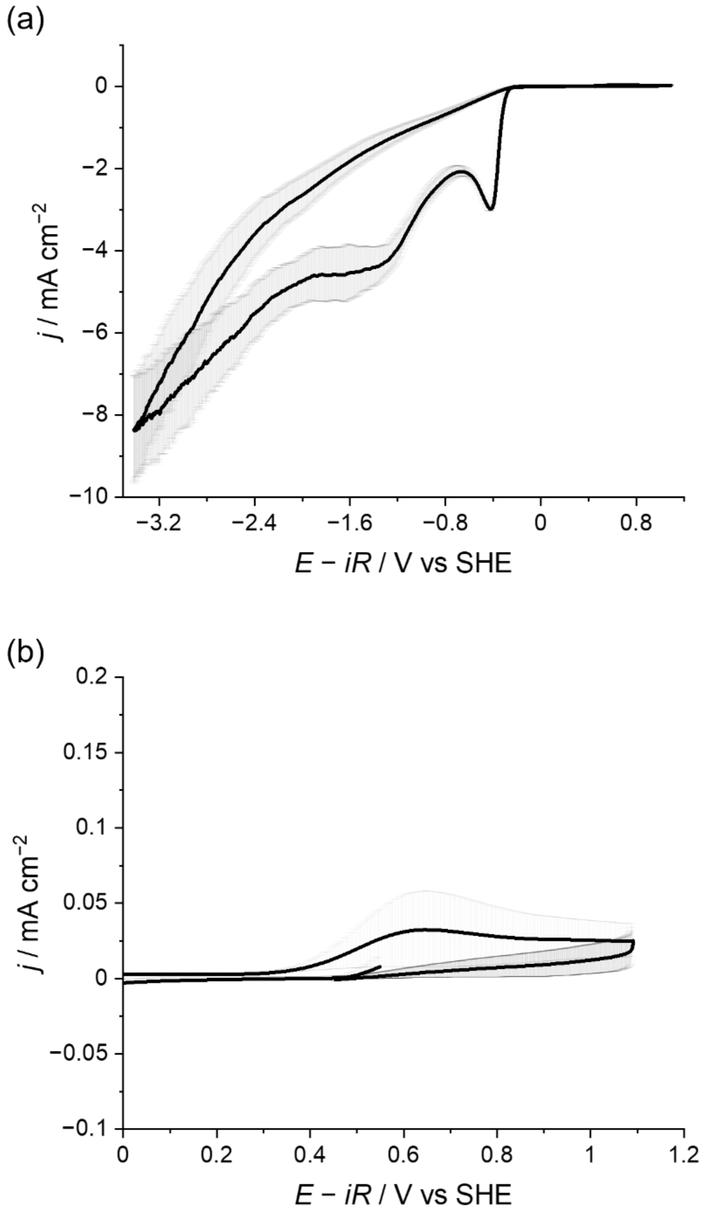

Figure S8. Graph showing the average and standard deviation of the results from 10 individual cyclic voltammograms obtained on Au electrode in CO_2-saturated 1 mM H_2SO_4 solution: (a) Full potential window, (b) Narrower potential window with a magnified *y*-axis scale. The electrode potential of individual voltammograms was initially scanned from the OCP to −3.4 V, then to +1.1 V, and back to the OCP at 50 mV s^{-1}. The non-zero starting current is a technical glitch arising from averaging 10 individual voltammograms whose OCP values are different between each other. The median curve of the 10 individual curves is shown in Figure 2 for comparison.

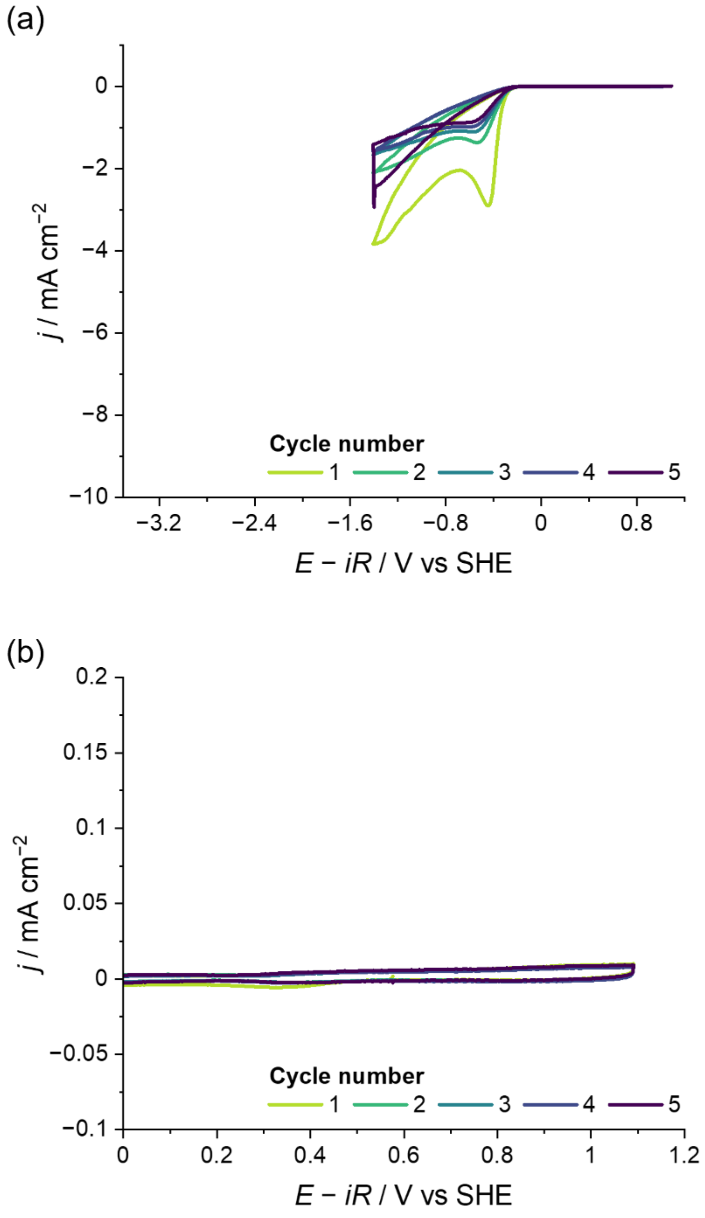

Figure S9. Cyclic voltammograms obtained on Au electrode in CO_2-saturated post-electrolysis 1 mM H_2SO_4 solution: (a) Full potential window, (b) Narrower potential window with a magnified *y*-axis scale. The pre-electrolysis was conducted to generate, if any, autogenous Au ions by scanning from the OCP to −3.4 V, then to +1.1 V, and back to the OCP at 50 mV s^{-1}. Using the post-electrolysis solution, the actual electroanalysis was conducted by scanning from the OCP to −1.4 V, then to +1.1 V, and back to the OCP at 50 mV s^{-1}. Comparison between this curve and a curve obtained in pristine electrolyte (cf. Figure 1, S1) suggests that the electroreduction of CO_2 is unlikely caused by autogenous Au ions.

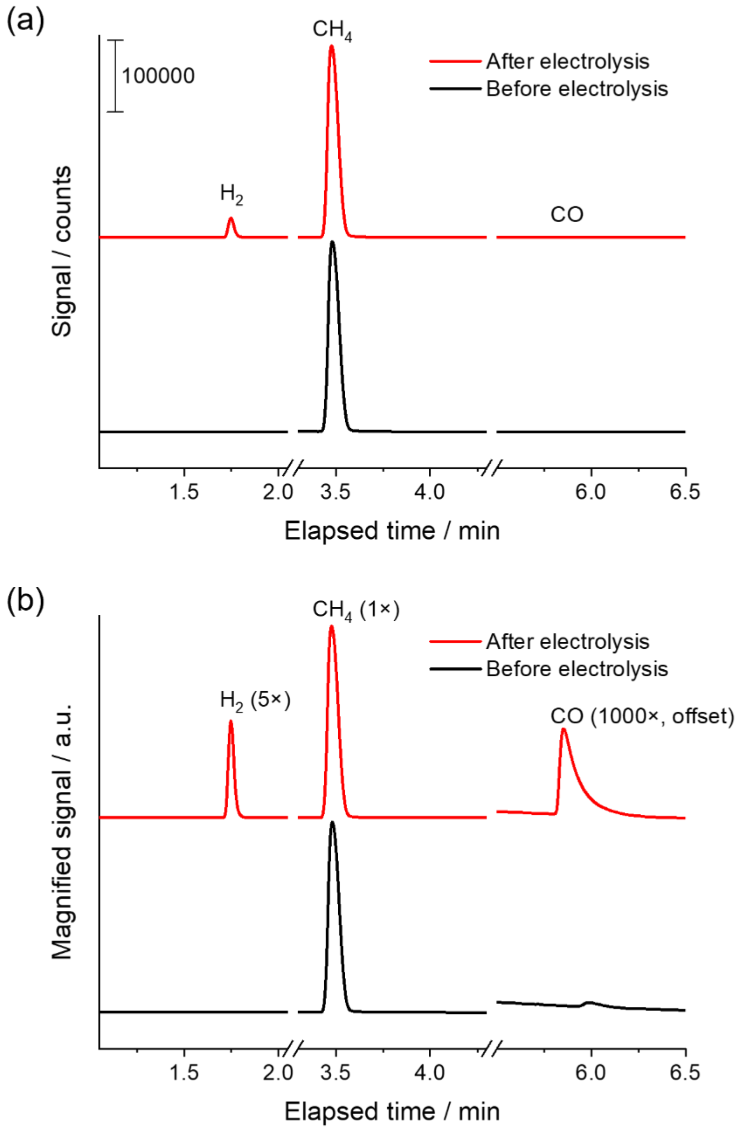

Figure S10. Gas chromatograms obtained before and after electrolysis from the headspace of the cell in the presence of 1% CH_4 as an internal standard. Electrolysis was conducted at −1.4 V for 1800 s with magnetic stirring at 600 rpm in CO_2-saturated 1 mM H_2SO_4 solution. (a) Actual signal. (b) Signals magnified from the actual signal (H_2 range: 5× magnified; CH_4 range: not magnified; CO_2 range: 1000× magnified).

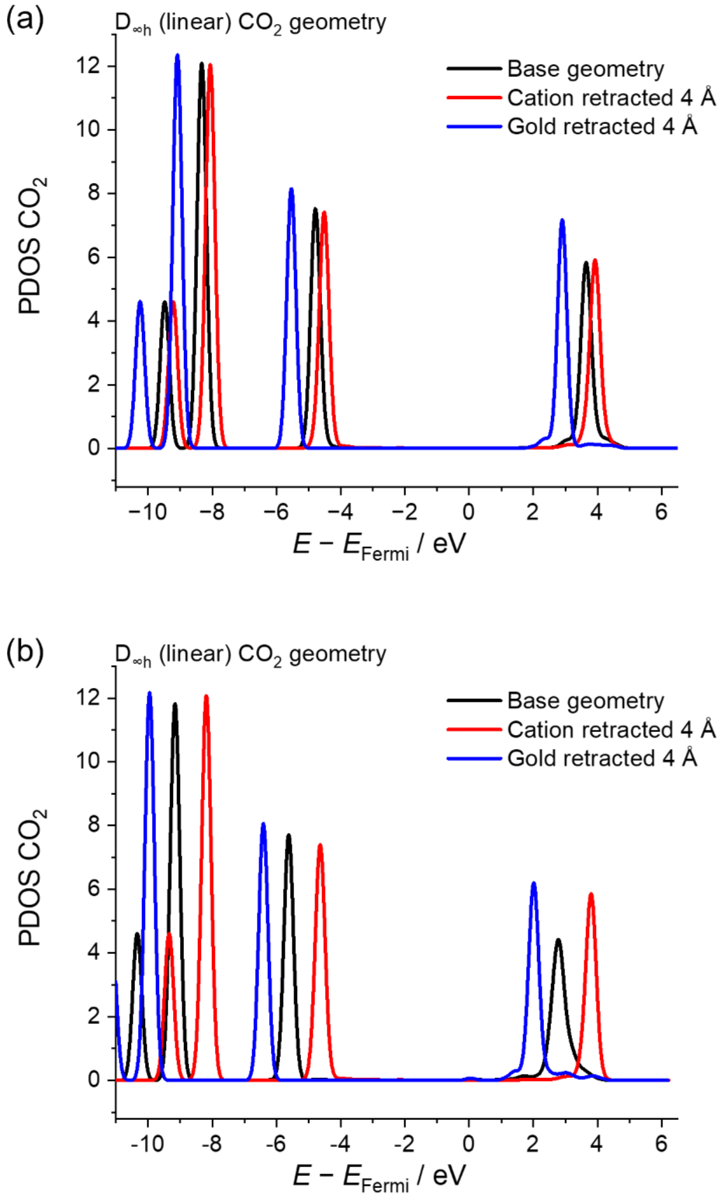

Figure S11. PDOS about the Fermi level of CO_2 physisorbed on Au(111) in the presence of (a) H_3O^+ and (b) K^+. Black trace: Gold slab at optimized distance for physisorption of CO_2 in the absence of cations. Red trace: Both CO_2 and Au(111) have been removed 4 Å from the cation in the cell *z*-direction, separating the cation from the system. Blue trace: Au(111) has been removed 4 Å from the CO_2 and cation, separating the Au from the system.

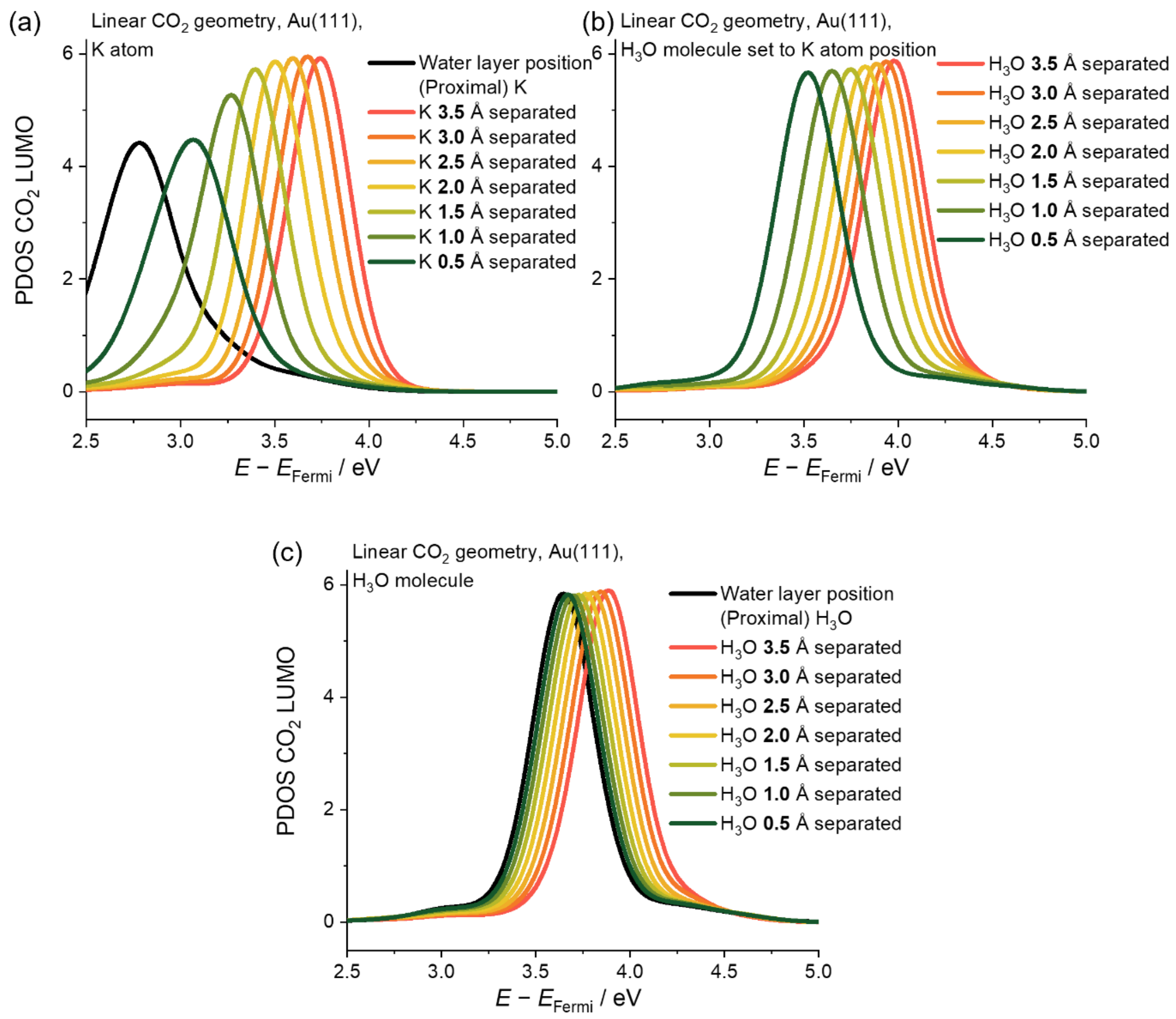

Figure S12. (a) Change in LUMO of CO_2 with the spatial retraction of K^+, displaying non-linear shift with separation in space and broadening of the LUMO PDOS peak indicative of CO_2 orbital hybridization with K^+; and (b) the same system but with H_3O^+ replacing K^+. (c) The test repeated, changing the H_3O^+ position from the relative position of the proximal K^+ ion in the K^+/water layer systems to the proximal H_3O^+ position from the H_3O^+/water layer system, indicating that the shift is largely electrostatic in nature.

(a)

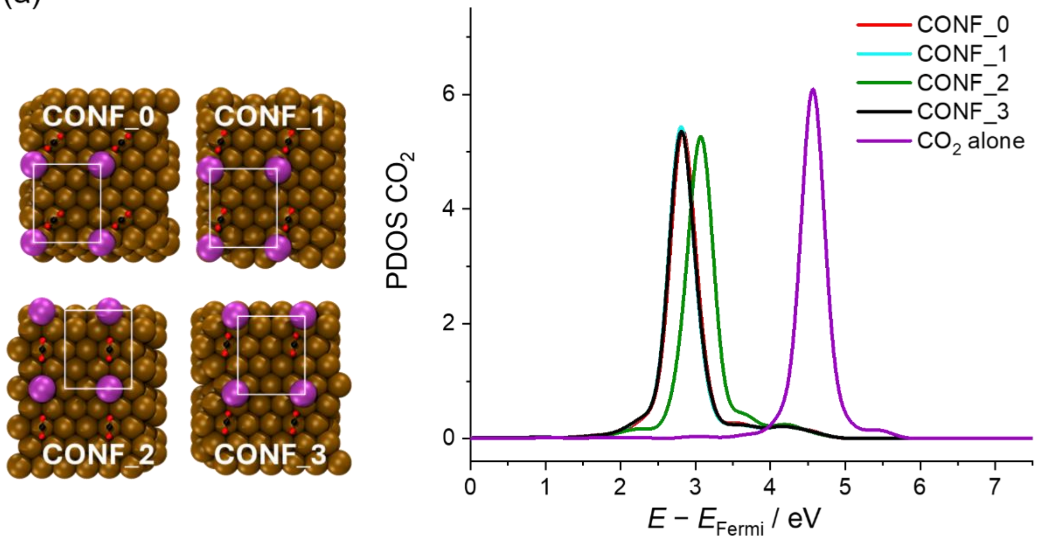

(b)

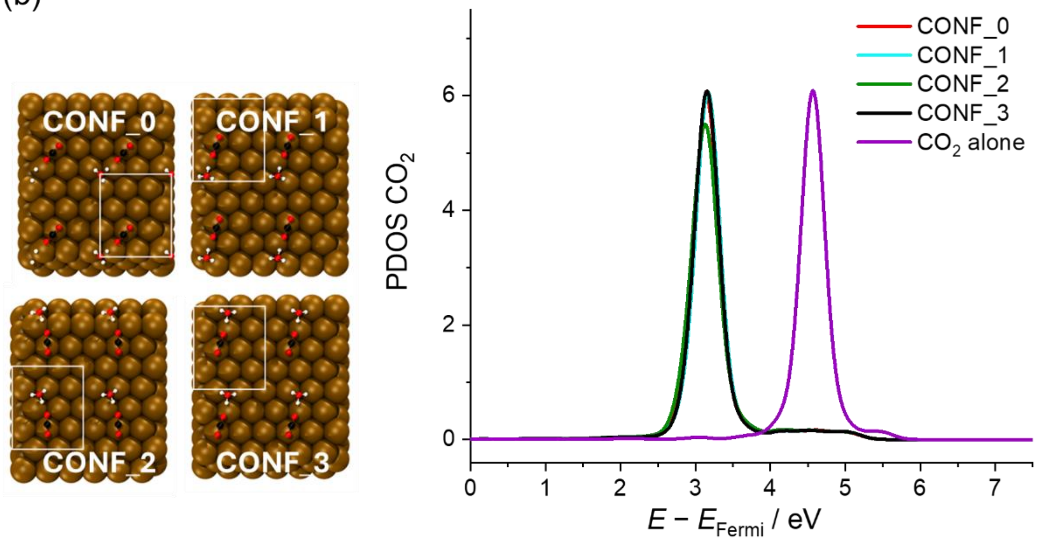

Figure S13. Full data set of geometry-relaxed configuration for (a) K^+- and (b) H_3O^+-co-adsorbed systems as referenced in Figure 4

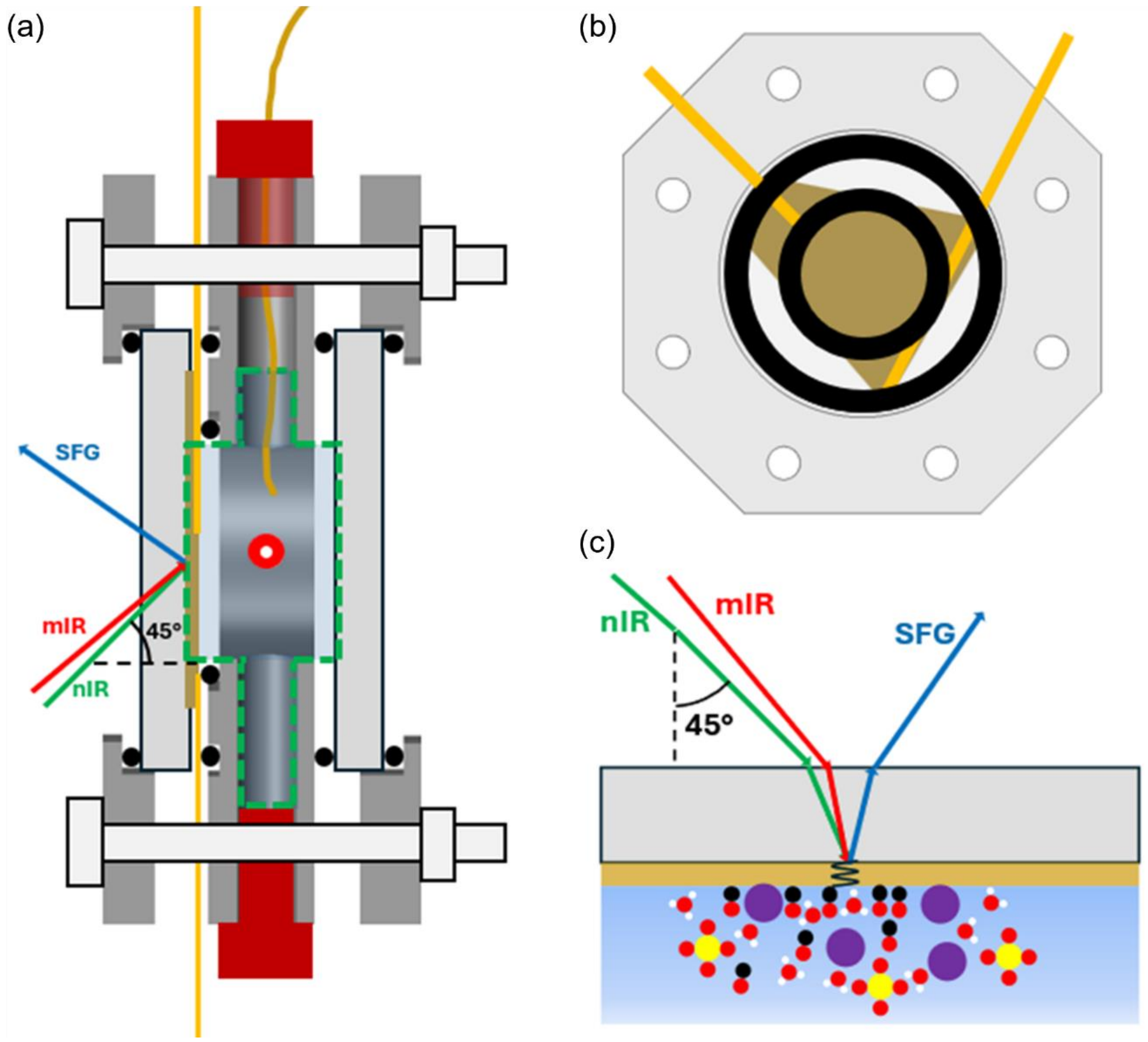

Figure S14. (a) A side cross-section of the VSFG cell and (b) the front view of the Au-coated window in the VSFG cell. The pieces of the cell (*dark gray*) are made from PEEK and are clamped together by screws and nuts (*white*). The CaF_2 windows (*gray*) are protected and sealed with NBR O-rings (*black*). The front window (*left-hand side in panel a*) is partially coated with a nanolayer of Au (*gold*) as the working electrode and is connected to the external circuit by two strips of Au foil (*yellow*). The Au counter electrode wire (*gold*) is fed through a rubber septum (*red*) in the top of the cell, while the leak-free Ag/AgCl reference electrode (*red with white center*) is fed through a rubber septum in the side of the cell. The volume occupied by electrolyte is indicated by the green dashed line. (c) VSFG optical geometry across the air/window, window/gold and gold/electrolyte interfaces.

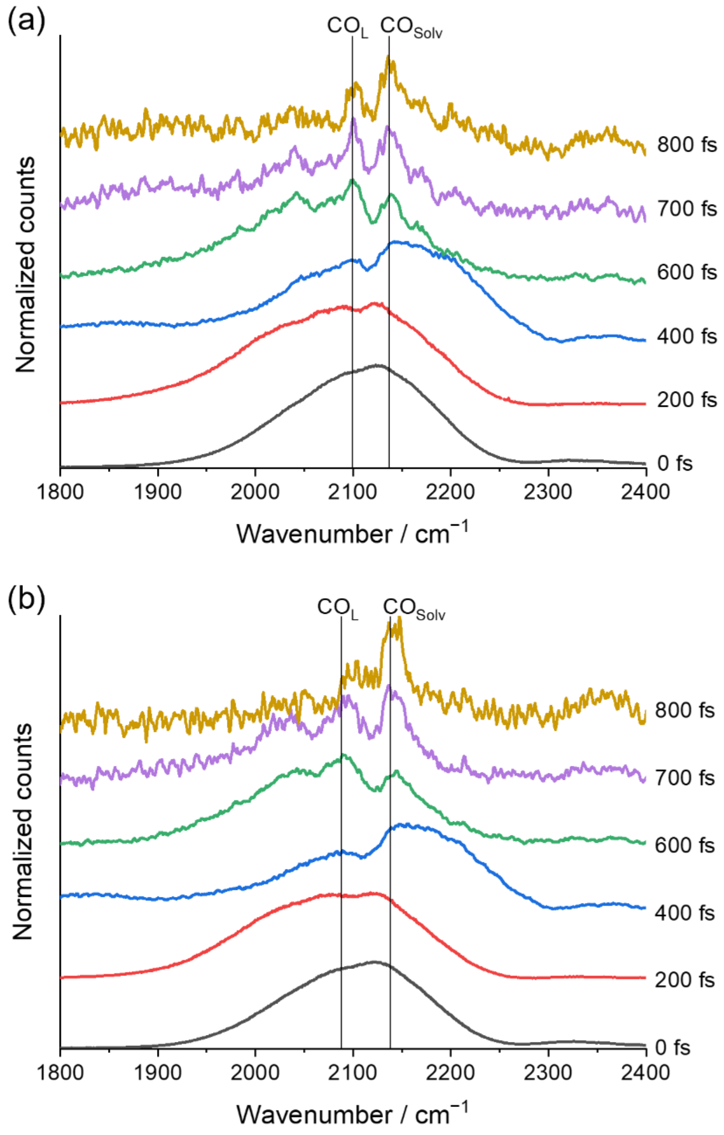

Figure S15. The peak-normalized VSFG spectra at −220 mV vs SHE at different nIR delay times for CO-purged (a) 0.1 M H_2SO_4 and (b) 0.1 M H_2SO_4 and 0.1 M K_2SO_4 solutions. The 600 fs, 700 fs and 800 fs spectra have been smoothed. The CO$_L$ and CO$_{Solv}$ bands have been highlighted for clarity.

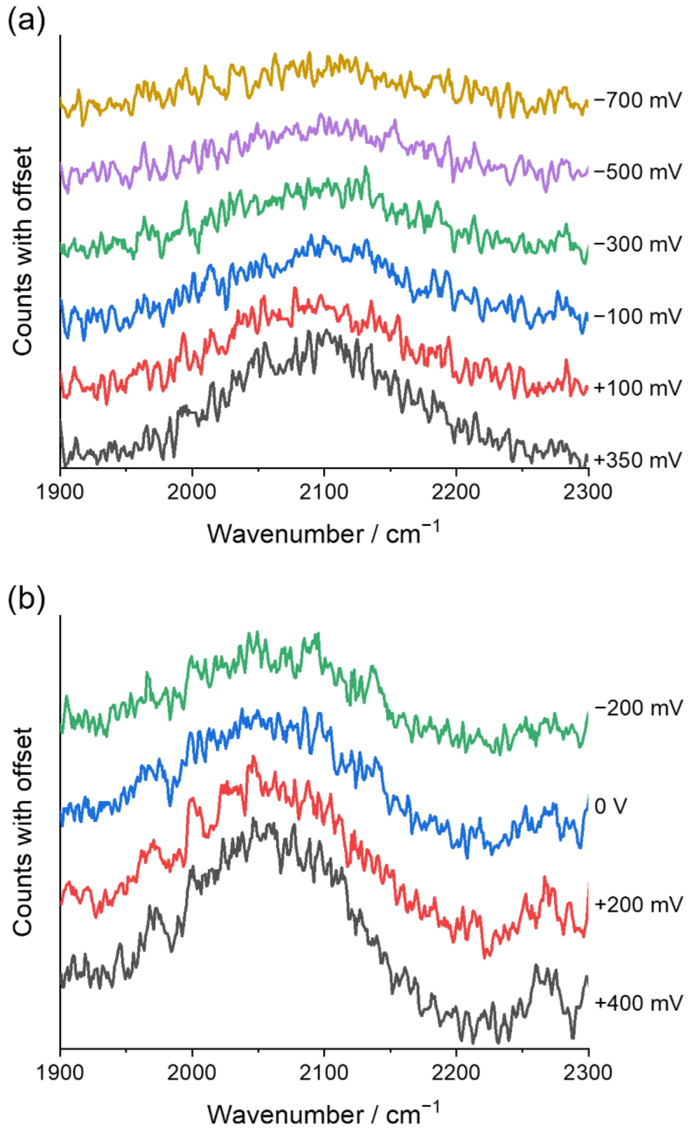

Figure S16. The VSFG spectra at different 800 fs nIR delay for Ar purged (a) 0.1 M H_2SO_4 and (b) 0.1 M H_2SO_4 and 0.1 M K_2SO_4 solutions at different applied potentials vs SHE.

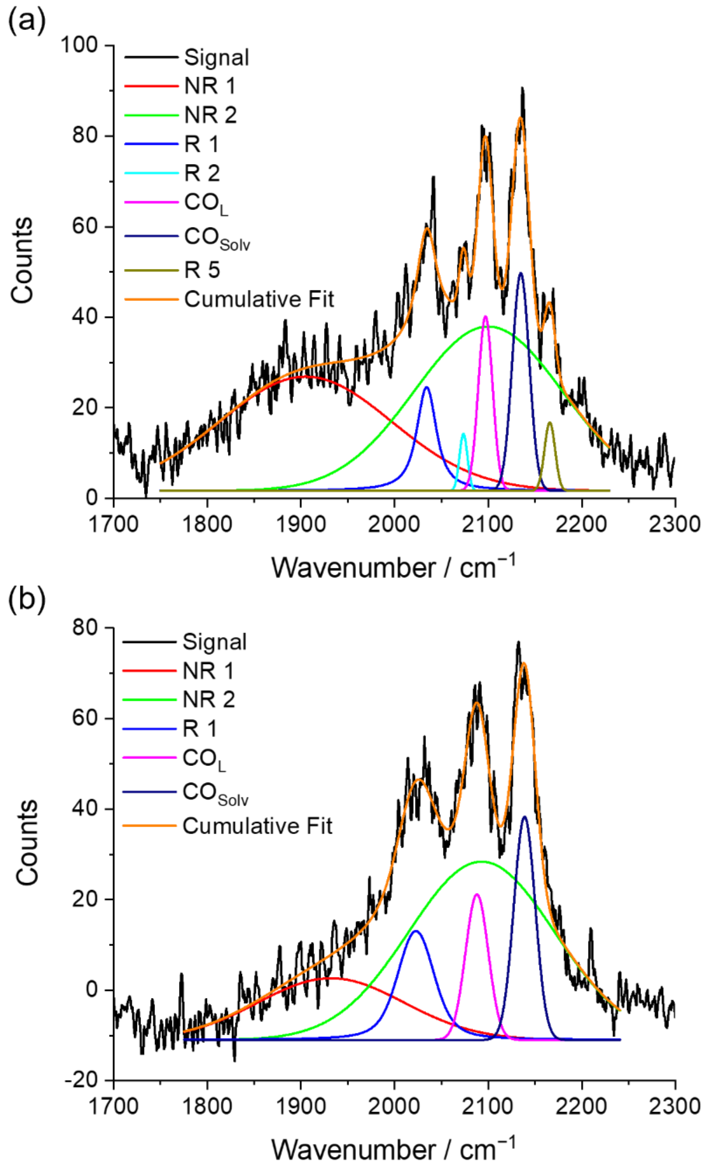

Figure S17. Smoothed VSFG spectra and their respective fits for CO purged (a) 0.1 M H_2SO_4 and (b) 0.1 M H_2SO_4 and 0.1 M K_2SO_4 solutions. Spectra were recorded at −220 mV vs SHE and with a 700 fs nIR delay. The signal in (a) is fit to 2 Gaussian curves (red and green) to fit for the non-resonant (NR) contribution to the signal and 5 Voigt curves to fit for the resonant (R) contributions to the signal. The signal in (b) is fit to 2 Gaussian curves (red and green, non-resonant) and 3 Voigt curves (resonant). CO_L and CO_{Solv} are the assigned fits attributed to CO linearly bound to Au, and solvated CO close to the electrode surface, respectively. R 1, R 2 and R 5 are other unassigned resonant peak fits relating to various unidentified CO environments.

Supplementary tables

Table S1. Inductively Coupled Plasma Mass Spectrometry Analysis of Electrolytes

	Li / ppb	Na / ppb	K / ppb	Cs / ppb	Au / ppb	Mg / ppb	Si / ppb
Control[a]	N/D[c]	N/D[c]	N/D[c]	N/D[c]	N/D[c]	N/D[c]	N/D[c]
−1.4 V[b]	N/D[c]	N/D[c]	N/D[c]	N/D[c]	N/D[c]	N/D[c]	4.003[d]
−1.8 V[b]	N/D[c]	N/D[c]	N/D[c]	N/D[c]	N/D[c]	N/D[c]	3.544[d]
−2.2 V[b]	N/D[c]	N/D[c]	N/D[c]	N/D[c]	N/D[c]	N/D[c]	1.724[d]
−2.6 V[b]	N/D[c]	N/D[c]	N/D[c]	N/D[c]	N/D[c]	N/D[c]	8.571[d]
−3.0 V[b]	N/D[c]	N/D[c]	N/D[c]	N/D[c]	N/D[c]	N/D[c]	3.681[d]
−3.4 V[b]	N/D[c]	N/D[c]	N/D[c]	N/D[c]	N/D[c]	N/D[c]	17.013

[a]Pristine electrolyte of 1 mM H_2SO_4; [b]Aliquot of 1 mM H_2SO_4 electrolyte after reaching the potential as indicated in the table (vs SHE); [c]Not detected as it is below the limit of detection (i.e. [Li] < 1 ppb, [Na] < 5 ppb, [K] < 5 ppb, [Cs] < 1 ppb, [Au] < 1 ppb, [Mg] < 1 ppb, [Si] < 10 ppb); [d]Detected as it is greater than 1 ppb but still remaining below the limit of detection of Si (i.e. 10 ppb).

Table S2. Calculated DFT Energies for the Configurations Labeled in Figure S13

System	Configuration	DFT Calculated Energy / eV
K^+	CONF_0	−151.0538
K^+	CONF_1	−151.0643
K^+	CONF_2	−150.9441
K^+	CONF_3	−151.0658
H_3O^+	CONF_0	−165.6245
H_3O^+	CONF_1	−165.6229
H_3O^+	CONF_2	−165.6026
H_3O^+	CONF_3	−165.6333

Supplementary references

(1) Chen, L. D.; Urushihara, M.; Chan, K.; Nørskov, J. K. Electric Field Effects in Electrochemical CO_2 Reduction. *ACS Catal.* **2016**, *6* (10), 7133-7139. DOI: 10.1021/acscatal.6b02299.

(2) Shin, S.-J.; Choi, H.; Ringe, S.; Won, D. H.; Oh, H.-S.; Kim, D. H.; Lee, T.; Nam, D.-H.; Kim, H.; Choi, C. H. A unifying mechanism for cation effect modulating C1 and C2 productions from CO_2 electroreduction. *Nat. Commun.* **2022**, *13* (1), 5482. DOI: 10.1038/s41467-022-33199-8.

(3) Ringe, S.; Clark, E. L.; Resasco, J.; Walton, A.; Seger, B.; Bell, A. T.; Chan, K. Understanding cation effects in electrochemical CO_2 reduction. *Energy Environ. Sci.* **2019**, *12* (10), 3001-3014. DOI: 10.1039/C9EE01341E.

(4) Hübner, J. L.; Lucchetti, L. E. B.; Nong, H. N.; Sharapa, D. I.; Paul, B.; Kroschel, M.; Kang, J.; Teschner, D.; Behrens, S.; Studt, F.; Knop-Gericke, A.; Siahrostami, S.; Strasser, P. Cation Effects on the Acidic Oxygen Reduction Reaction at Carbon Surfaces. *ACS Energy Lett.* **2024**, *9* (4), 1331-1338. DOI: 10.1021/acsenergylett.3c02743.

(5) Bender, J. T.; Sanspeur, R. Y.; Valles, A. E.; Uvodich, A. K.; Milliron, D. J.; Kitchin, J. R.; Resasco, J. The Potential of Zero Total Charge Predicts Cation Effects for the Oxygen Reduction Reaction. *ACS Energy Lett.* **2024**, *9* (9), 4724-4733. DOI: 10.1021/acsenergylett.4c01897.

(6) Qin, H.-G.; Li, F.-Z.; Du, Y.-F.; Yang, L.-F.; Wang, H.; Bai, Y.-Y.; Lin, M.; Gu, J. Quantitative Understanding of Cation Effects on the Electrochemical Reduction of CO_2 and H^+ in Acidic Solution. *ACS Catalysis* **2023**, *13* (2), 916-926. DOI: 10.1021/acscatal.2c04875.

(7) Becke, A. D. Density-functional exchange-energy approximation with correct asymptotic behavior. *Phys. Rev. A* **1988**, *38* (6), 3098-3100. DOI: 10.1103/PhysRevA.38.3098.

(8) Gardner, A. M.; Saeed, K. H.; Cowan, A. J. Vibrational sum-frequency generation spectroscopy of electrode surfaces: studying the mechanisms of sustainable fuel generation and utilisation. *Phys. Chem. Chem. Phys.* **2019**, *21* (23), 12067-12086. DOI: 10.1039/C9CP02225B.

(9) Wain, A. J.; O'Connell, M. A. Advances in surface-enhanced vibrational spectroscopy at electrochemical interfaces. *Adv. Phys. X* **2017**, *2* (1), 188-209. DOI: 10.1080/23746149.2016.1268931.

(10) Sorenson, S. A.; Patrow, J. G.; Dawlaty, J. M. Solvation Reaction Field at the Interface Measured by Vibrational Sum Frequency Generation Spectroscopy. *J. Am. Chem. Soc.* **2017**, *139* (6), 2369-2378. DOI: 10.1021/jacs.6b11940.

(11) Levinson, N. M.; Fried, S. D.; Boxer, S. G. Solvent-Induced Infrared Frequency Shifts in Aromatic Nitriles Are Quantitatively Described by the Vibrational Stark Effect. *J. Phys. Chem. B* **2012**, *116* (35), 10470-10476. DOI: 10.1021/jp301054e.

(12) Sarkar, S.; Maitra, A.; Banerjee, S.; Thoi, V. S.; Dawlaty, J. M. Electric Fields at Metal–Surfactant Interfaces: A Combined Vibrational Spectroscopy and Capacitance Study. *J. Phys. Chem. B* **2020**, *124* (7), 1311-1321. DOI: 10.1021/acs.jpcb.0c00560.

(13) Bhattacharyya, D.; Videla, P. E.; Cattaneo, M.; Batista, V. S.; Lian, T.; Kubiak, C. P. Vibrational Stark shift spectroscopy of catalysts under the influence of electric fields at electrode–solution interfaces. *Chem. Sci.* **2021**, *12* (30), 10131-10149. DOI: 10.1039/D1SC01876K.

(14) Zhu, Q.; Wallentine, S. K.; Deng, G.-H.; Rebstock, J. A.; Baker, L. R. The Solvation-Induced Onsager Reaction Field Rather than the Double-Layer Field Controls CO_2 Reduction on Gold. *JACS Au* **2022**, *2* (2), 472-482. DOI: 10.1021/jacsau.1c00512.

(15) Rebstock, J. A.; Zhu, Q.; Baker, L. R. Exploring the influence of interfacial solvation on electrochemical CO_2 reduction using plasmon-enhanced vibrational sum frequency generation spectroscopy. *ChemCatChem* **2024**, *16* (14), e202301301. DOI: 10.1002/cctc.202301301.

(16) Gardner, A. M.; Neri, G.; Siritanaratkul, B.; Jang, H.; Saeed, K. H.; Donaldson, P. M.; Cowan, A. J. Potential Dependent Reorientation Controlling Activity of a Molecular Electrocatalyst. *J. Am. Chem. Soc.* **2024**, *146* (11), 7130-7134. DOI: 10.1021/jacs.3c13076.

(17) Gunathunge, C. M.; Li, J.; Li, X.; Waegele, M. M. Surface-Adsorbed CO as an Infrared Probe of Electrocatalytic Interfaces. *ACS Catal.* **2020**, *10* (20), 11700-11711. DOI: 10.1021/acscatal.0c03316.

(18) Rebstock, J. A.; Zhu, Q.; Baker, L. R. Comparing interfacial cation hydration at catalytic active sites and spectator sites on gold electrodes: understanding structure sensitive CO_2 reduction kinetics. *Chem. Sci.* **2022**, *13* (25), 7634-7643. DOI: 10.1039/D2SC01878K.

(19) Patrow, J. G.; Sorenson, S. A.; Dawlaty, J. M. Direct Spectroscopic Measurement of Interfacial Electric Fields near an Electrode under Polarizing or Current-Carrying Conditions. *J. Phys. Chem. C* **2017**, *121* (21), 11585-11592. DOI: 10.1021/acs.jpcc.7b03134.

(20) Pastor, E.; Lian, Z.; Xia, L.; Ecija, D.; Galán-Mascarós, J. R.; Barja, S.; Giménez, S.; Arbiol, J.; López, N.; García de Arquer, F. P. Complementary probes for the electrochemical interface. *Nat. Rev. Chem.* **2024**, *8* (3), 159-178. DOI: 10.1038/s41570-024-00575-5.

(21) Lagutchev, A.; Hambir, S. A.; Dlott, D. D. Nonresonant Background Suppression in Broadband Vibrational Sum-Frequency Generation Spectroscopy. *J. Phys. Chem. C* **2007**, *111* (37), 13645-13647. DOI: 10.1021/jp075391j.

(22) Lambert, A. G.; Davies, P. B.; Neivandt, D. J. Implementing the Theory of Sum Frequency Generation Vibrational Spectroscopy: A Tutorial Review. *Appl. Spectrosc. Rev.* **2005**, *40* (2), 103-145. DOI: 10.1081/ASR-200038326.

(23) Banerji, L. C.; Jang, H.; Gardner, A. M.; Cowan, A. J. Studying the cation dependence of CO_2 reduction intermediates at Cu by in situ VSFG spectroscopy. *Chem. Sci.* **2024**, *15* (8), 2889-2897. DOI: 10.1039/D3SC05295H.

(24) Deng, G.-H.; Zhu, Q.; Rebstock, J.; Neves-Garcia, T.; Baker, L. R. Direct observation of bicarbonate and water reduction on gold: understanding the potential dependent proton source during hydrogen evolution. *Chem. Sci.* **2023**, *14* (17), 4523-4531. DOI: 10.1039/D3SC00897E.

(25) Wallentine, S.; Bandaranayake, S.; Biswas, S.; Baker, L. R. Plasmon-Resonant Vibrational Sum Frequency Generation of Electrochemical Interfaces: Direct Observation of Carbon Dioxide Electroreduction on Gold. *J. Phys. Chem. A* **2020**, *124* (39), 8057-8064. DOI: 10.1021/acs.jpca.0c04268.

(26) Wallentine, S.; Bandaranayake, S.; Biswas, S.; Baker, L. R. Direct Observation of Carbon Dioxide Electroreduction on Gold: Site Blocking by the Stern Layer Controls CO_2 Adsorption Kinetics. *J. Phys. Chem. Lett.* **2020**, *11* (19), 8307-8313. DOI: 10.1021/acs.jpclett.0c02628.

(27) Sun, S.-G.; Cai, W.-B.; Wan, L.-J.; Osawa, M. Infrared Absorption Enhancement for CO Adsorbed on Au Films in Perchloric Acid Solutions and Effects of Surface Structure Studied by Cyclic Voltammetry, Scanning Tunneling Microscopy, and Surface-Enhanced IR Spectroscopy. *J. Phys. Chem. B* **1999**, *103* (13), 2460-2466. DOI: 10.1021/jp984028x.

(28) Miyake, H.; Ye, S.; Osawa, M. Electroless deposition of gold thin films on silicon for surface-enhanced infrared spectroelectrochemistry. *Electrochem. Commun.* **2002**, *4* (12), 973-977. DOI: 10.1016/S1388-2481(02)00510-6.

(29) Dunwell, M.; Wang, J.; Yan, Y.; Xu, B. Surface enhanced spectroscopic investigations of adsorption of cations on electrochemical interfaces. *Phys. Chem. Chem. Phys.* **2017**, *19* (2), 971-975. DOI: 10.1039/C6CP07207K.

(30) Wuttig, A.; Yaguchi, M.; Motobayashi, K.; Osawa, M.; Surendranath, Y. Inhibited proton transfer enhances Au-catalyzed CO_2-to-fuels selectivity. *Proc. Natl. Acad. Sci. U.S.A.* **2016**, *113* (32), E4585-E4593. DOI: 10.1073/pnas.1602984113.

(31) Barclay, A. J.; Pietropolli Charmet, A.; McKellar, A. R. W.; Moazzen-Ahmadi, N. Exploring the next step in micro-solvation of CO in water: Infrared spectra and structural calculations of $(H_2O)_4$–CO and $(D_2O)_4$–CO. *J. Chem. Phys.* **2021**, *154* (4), 044310. DOI: 10.1063/5.0038188.

(32) Chen, D.-J.; Allison, T. C.; Tong, Y. J. Mechanistic Insights into Electro-Oxidation of Solution CO on the Polycrystalline Gold Surface as Seen by in Situ IR Spectroscopy. *J. Phys. Chem. C* **2016**, *120* (29), 16132-16139. DOI: 10.1021/acs.jpcc.6b00024.
